# Analytical continuation of two-dimensional wave fields

**DOI:** 10.1098/rspa.2020.0681

**Published:** 2021-01-06

**Authors:** Raphaël C. Assier, Andrey V. Shanin

**Affiliations:** 1 Department of Mathematics, The University of Manchester, Oxford Road, Manchester M13 9PL, UK; 2 Department of Physics (Acoustics Division), Moscow State University, Leninskie Gory, 119992 Moscow, Russia

**Keywords:** diffraction, analytical continuation, multi-variable complex analysis

## Abstract

Wave fields obeying the two-dimensional Helmholtz equation on branched surfaces (Sommerfeld surfaces) are studied. Such surfaces appear naturally as a result of applying the reflection method to diffraction problems with straight scatterers bearing ideal boundary conditions. This is for example the case for the classical canonical problems of diffraction by a half-line or a segment. In the present work, it is shown that such wave fields admit an analytical continuation into the domain of two complex coordinates. The branch sets of such continuation are given and studied in detail. For a generic scattering problem, it is shown that the set of all branches of the multi-valued analytical continuation of the field has a finite basis. Each basis function is expressed explicitly as a Green’s integral along so-called double-eight contours. The finite basis property is important in the context of coordinate equations, introduced and used by the authors previously, as illustrated in this article for the particular case of diffraction by a segment.

## 1. Introduction

In this paper we study two-dimensional diffraction problems for the Helmholtz equation belonging to a special class: namely those that can be reformulated as problems of propagation on branched surfaces with finitely many sheets. At least two classical canonical diffraction problems belong to this class: the Sommerfeld problem of diffraction by a half-line with ideal boundary conditions, and the problem of diffraction by an ideal segment. There are also some other important problems belonging to this class, they are listed in appendix A. The branched surface for such a problem is referred to as a *Sommerfeld surface* and denoted by *S*. This surface has several sheets over the real Cartesian plane (*x*_1_, *x*_2_), and these sheets are connected at several branch points.

The solution of the corresponding problem is denoted by *u*(*x*_1_, *x*_2_) and is assumed to be known on *S*. We consider the possibility to continue the solution *u* into the complex domain of the coordinates $(x_{1},x_{2})\in C^{2}$. Namely, we are looking for a function *u*_c_(*x*_1_, *x*_2_) which is analytical almost everywhere, obeys the complex Helmholtz equation, and is equal to *u*(*x*_1_, *x*_2_) for real (*x*_1_, *x*_2_).

It is shown in the paper that such a continuation can be constructed using Green’s third identity. The integration contours used involve rather complicated loops drawn on *S*, referred to as double-eight or Pochhammer contours. The integrand comprises the function *u* on *S*, a complexified kernel and their first derivatives.

The analytical continuation *u*_c_ is a branched (i.e. multi-valued) function in $C^{2}$. Its branch set *T* is the union of the complex characteristics of the Helmholtz equation passing through the branch points of *S*. At each point of $C^{2}$ not belonging to the branch set *T* there exists an infinite number of branches of *u*_c_, i.e. infinitely many possible values of *u*_*c*_ in the neighbourhood of this point. However, we prove here that these branches have a *finite basis*, such that any branch can be expressed as a linear combination of a finite number of *basis functions* with integer coefficients.

This property of the analytical continuation is an important property of the initial (real) diffraction problem. Namely, it indicates that one can build the so-called *coordinate equations*, which are ‘multidimensional ordinary differential equations’ [1–3]. Thus, a PDE becomes effectively solved as a finite set of ODEs.

To emphasize the non-triviality of the statements proven in this paper and the importance of studying the analytical continuation of the field, we can say that we have not yet succeeded in generalizing the results to the case of three-dimensional diffraction problems. It is possible to build a three-dimensional analogue of Sommerfeld surfaces (for example, it can be done for the ideal quarter-plane diffraction problem), but at the moment it does not seem possible to show that the analytical continuation possesses a finite basis. Thus, a generalization of the coordinate equations seems not to be possible for three-dimensional problems. In the future, with the quarter-plane problem in mind, we wish to extend these results to wave propagation problems involving the Laplace–Beltrami operator defined on the surface of a sphere with a cut (see e.g. [4–6] for more detail on the links between Laplace–Beltrami and the quarter-plane problem).

The ideas behind this work were inspired in part by our recent investigations of applications of multi-variable complex analysis to diffraction problems [7–11], in part by our work on coordinate equations [1–5] and in part by the work of Sternin and his co-authors [12–14], in which the analytical continuation of wave fields is considered.

In [12–14], the practical problems of finding minimal configurations of sources producing certain fields, or continuation of fields through complicated boundaries, are solved.

Different techniques can be used to study the analytical continuation of a wave field. We are using Green’s theorem as in [12,15]. Alternatives include the use of Radon transforms [12,14], series representations or Schwarz’s reflection principle.

The rest of the paper is organized as follows. In §2 we define the concept of a Sommerfeld surface and introduce the notion of diffraction problems on such an object. In §3, we specify what we mean by the analytical continuation of a wave field *u* to $C^{2}$, and discuss the notion of branching of functions of two complex variables. In §4, we give an integral representation that enables us to analytically continue *u* from a point in *S* to a point in $C^{2}$. The obtained function *u*_*c*_ is multi-valued in $C^{2}$, and, in §5, we study in detail its branching structure and specify all its possible branches by means of Green’s integrals involving double-eight contours. In particular, we show that there exists a finite basis of functions such that any branch of the analytical continuation can be expressed as a linear combination of these basis functions with integer coefficients. In §6, we apply the general theory developed thus far to the specific problem of diffraction by an ideal strip, showing that, in this case, the number of basis functions can be reduced to four; we describe explicitly and constructively, via some matrix algebra, all possible branches of the analytical continuation. Finally, in §7, still for the strip problem, we show that our results imply the existence of the so-called coordinate equations, effectively reducing the diffraction problem to a set of two multi-dimensional ODEs.

## 2. A diffraction problem on a real Sommerfeld surface

Let us start by defining more precisely the concept of a Sommerfeld surface. Take *M* copies of the plane $R^{2}$ (called *sheets* of the surface), each equipped with the Cartesian coordinates (*x*_1_, *x*_2_). Let there exist *N* affixes of branch points $P_{1},\ldots ,P_{N}\in R^{2}$ with coordinates $\left(X_{1}^{\left(j\right)},X_{2}^{\left(j\right)}\right)$, *j* = 1, …, *N*. Consider a set of non-intersecting cuts on each sheet, connecting the points *P*_*j*_ with each other or with infinity. Finally, let the sides of the cuts be connected (‘glued’) to each other according to an arbitrary scheme. The connection of the sides should obey the following rules: (a) only points having the same coordinates (*x*_1_, *x*_2_) can be glued to each other; (b) one can glue a single ‘left’ side of a cut to a single ‘right’ side of this cut on another sheet; and (c) a side of a cut should be glued to a side of another cut as a whole.

**Figure 1.**
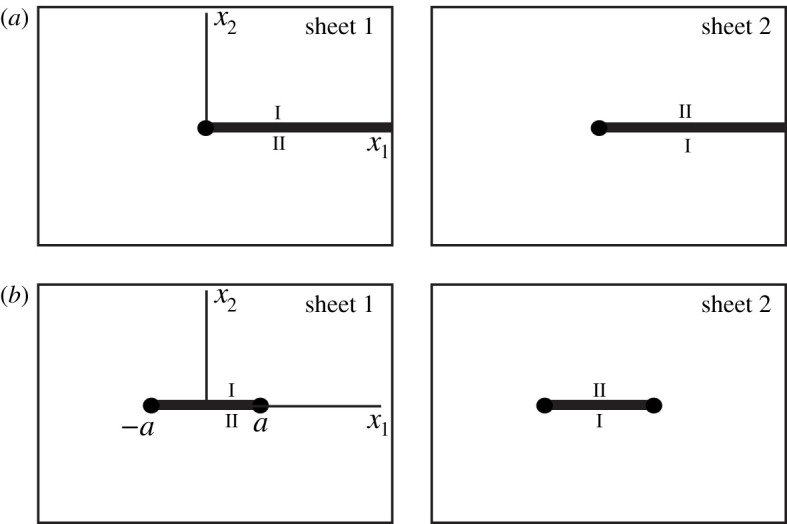
Sommerfeld surfaces for the two-dimensional problems of the Dirichlet half-line (*a*), and the Dirichlet segment (*b*). The cuts are shown by thick lines. The sides of the cuts glued to each other bear the same Roman number. The associated branch points are denoted by black discs.

Upon allowing spurious cuts, i.e. cuts glued to themselves, it is possible for the set of cuts to be the same on all sheets. The result of assembling the sheets is a Sommerfeld surface denoted by *S*. We assume everywhere that *N* and *M* are finite integers. Two examples of Sommerfeld surfaces are shown in figure 1.

The concept of Sommerfeld surfaces is naturally very close to that of Riemann surfaces of analytic functions of a complex variable. For example, the Sommerfeld surfaces shown in figure 1 can be treated as the Riemann surfaces of the functions $\sqrt{x_{1}+ix_{2}}$ and $\sqrt{{\left(x_{1}+ix_{2}\right)}^{2}-a^{2}}$, respectively. However, we prefer to refer to them as Sommerfeld surfaces since the coordinates *x*_1_ and *x*_2_ are real here, and since we would like to avoid confusion with the complex context that will be developed below.

Sommerfeld surfaces emerge naturally when the reflection method is applied to a two-dimensional diffraction problem with straight ideal boundaries. For example, the surfaces shown in figure 1 help one to solve the classical Sommerfeld problem of diffraction by a Dirichlet half-line [16] and the problem of diffraction by a Dirichlet segment [1]. A connection between the diffraction problems and the Sommerfeld surfaces is discussed in more details in appendix A, where the class of scatterers leading to finite-sheeted Sommerfeld surfaces is described.

**Figure 2.**
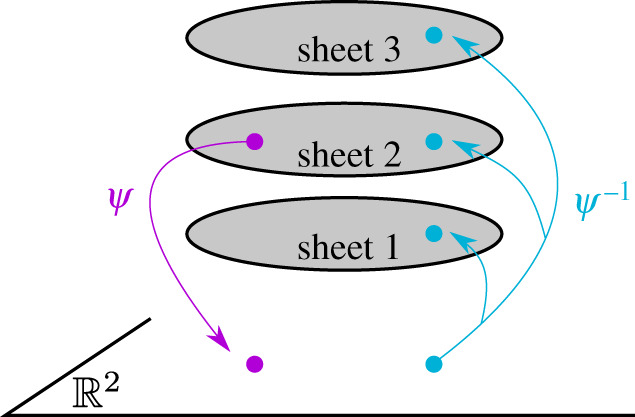
Diagrammatic illustration of the natural projection *ψ* of a Sommerfeld surface *S* with *M* = 3. (Online version in colour.)

There exists a natural projection *ψ* of a Sommerfeld surface *S* to $R^{2}$. For any small neighbourhood $U\subset R^{2}$ not including any of the branch points *P*_*j*_ the pre-image *ψ*^−1^ (*U*) is a set of *M* samples of *U*, as illustrated in figure 2.

Let *u* be a continuous single-valued function on some Sommerfeld surface *S* (thus, *u* is generally an *M*-valued function on $R^{2}$). For any neighbourhood $U\subset R^{2}\setminus \{P_{1},\ldots ,P_{N}\}$, let *u* obey the (real) Helmholtz equation

2.1 $$(\partial_{x_{1}}^{2}+\partial_{x_{2}}^{2}+k^{2})u(x_{1},x_{2})=0,$$

on each sample of *ψ*^−1^ (*U*). The wavenumber parameter k is chosen to have a positive real part and a vanishingly small positive imaginary part mimicking damping of waves.

Let *u* also obey the Meixner condition at the branch points *P*_*j*_. The Meixner condition characterizes the local finiteness of the energy-type integral

$$\iint (|\nabla u{|}^{2}+|u{|}^{2})\;\text{d}x_{1}dx_{2},$$

near a branch point. In particular, it guarantees the absence of sources at the branch points of *S*.

A function *u* obeying the equation (2.1) in the sense explained above and the Meixner condition will be referred to as a function obeying the Helmholtz equation on *S*.

Let us now formulate a diffraction problem on a Sommerfeld surface *S*: find a function *u* obeying the Helmholtz equation on *S* such that, on the physical sheet, *u* can be represented as a sum

2.2 $$u=u^{\text{in}}+u^{\text{sc}},\;\text{where}\;u^{\text{in}}=\exp\{-ik(x_{1}\cos\varphi^{\text{in}}+x_{2}\sin\varphi^{\text{in}})\}$$

is a known plane wave incident field. Here φ^in^ is the angle of incidence, and an appropriate radiation condition is imposed. Neither *u*^in^ nor *u*^sc^ are continuous, but their sum is. The field *u* is then constructed on the other sheets of *S* by means of the reflection principles as explained in appendix A. The resulting field *u* satisfies the Helmholtz equation on *S* and is regular everywhere apart from a finite number of branch points.

In the rest of the paper, we assume that the existence and uniqueness theorem is proven for the chosen *S* and k and that the field *u* is fully known on *S*.

## 3. Analytical continuation of the field and its branching

Our aim is to build an analytical continuation *u*_c_(*x*_1_, *x*_2_) of the solution *u*(*x*_1_, *x*_2_) of a certain diffraction problem on a Sommerfeld surface *S*. The continuation has the following sense.

Let *x*_1_ and *x*_2_ be complex variables, i.e. $(x_{1},x_{2})\in C^{2}$. Naturally, $C^{2}$ is a space of real dimension 4. Let $T\subset C^{2}$ be a singularity set (built below), which is a union of several manifolds of real dimension 2. Let *T* be such that the intersection of *T* with the real plane $R^{2}\subset C^{2}$ is the set of branch points *P*_1_, …, *P*_*N*_.

The continuation *u*_c_ should obey three conditions.

— The continuation *u*_c_(*x*_1_, *x*_2_) should be a multi-valued analytical function on $C^{2}\setminus T$. Each branch of *u*_c_ in any small domain $U\subset C^{2}$ not intersecting with *T* should obey the Cauchy–Riemann conditions
  3.1 $${\bar{\partial }}_{x_{\ell }}u_{\text{c}}=0,\;\text{where}\;{\bar{\partial }}_{x_{\ell }}\equiv \frac{1}{2}\left(\frac{\partial }{\partial \text{Re}[x_{\ell }]}+i\frac{\partial }{\partial \text{Im}[x_{\ell }]}\right),\;\ell \in \{1,2\}.$$
— The continuation *u*_c_(*x*_1_, *x*_2_) should obey the complex Helmholtz equation in $C^{2}\setminus T$:
  3.2 $$(\partial_{x_{1}}^{2}+\partial_{x_{2}}^{2}+k^{2})u_{\text{c}}=0,\;\text{where}\;\partial_{x_{\ell }}\equiv \frac{1}{2}\left(\frac{\partial }{\partial \text{Re}[x_{\ell }]}-i\frac{\partial }{\partial \text{Im}[x_{\ell }]}\right),\;\ell \in \{1,2\}.$$
  Note that the notation $\partial_{x_{\ell }}$ is used both in the real (2.1) and in the complex (3.2) context. However one can see that if the Cauchy–Riemann conditions are valid, the complex derivative gives just the same result as the real one.^1^
— When considering the restriction of *u*_c_(*x*_1_, *x*_2_) onto $R^{2}$, over each point of $R^{2}\setminus \{P_{1},\ldots P_{N}\}$, there should exist *M* branches of *u*_c_ equal to the values of *u* on *S*.

Let us describe, without a proof, the structure of the singularity set *T* and the branching structure of *u*_c_. Later on, we shall build *u*_c_ explicitly, and one will be able to check the correctness of these statements.

The branch points *P*_*j*_ are singularities of the field *u* on the real plane. According to the general theory of partial differential equations, the singularities propagate along the characteristics of the PDE (the Helmholtz equation here). Thus, it is natural to expect that *T* is the union of the characteristics passing through the points $P_{j}=(X_{1}^{\left(j\right)},X_{2}^{\left(j\right)})\in R^{2}$. Since the Helmholtz equation is elliptic, these characteristics are complex. They are given for *j* ∈ 1, …, *N* by

3.3 $$L_{1}^{\left(j\right)}=\{(x_{1},x_{2})\in C^{2},x_{1}+ix_{2}=X_{1}^{\left(j\right)}+iX_{2}^{\left(j\right)}\}$$

and

3.4 $$L_{2}^{\left(j\right)}=\{(x_{1},x_{2})\in C^{2},x_{1}-ix_{2}=X_{1}^{\left(j\right)}-iX_{2}^{\left(j\right)}\}.$$

One can see that $L_{1,2}^{\left(j\right)}$ are complex lines having real dimension 2. We will hence refer to them as *2-lines*. Their intersection with $R^{2}$ is the set of the points *P*_*j*_, i.e. $L_{1}^{\left(j\right)}\cap L_{2}^{\left(j\right)}=P_{j}$. It is interesting to note that topologically, the 2-lines $L_{1,2}^{\left(j\right)}$ are similar to the *complex rays* considered in [17], in the sense that they are both two-dimensional manifolds embedded in $C^{2}$, though the main difference between the two is that complex rays are complexified characteristics of the eikonal equation, while the branch 2-lines here are the characteristics of the Helmholtz equation.

We demonstrate below that the 2-lines $L_{1,2}^{\left(j\right)}$ are, generally, branch 2-lines of *u*_c_. The branching of functions of several complex variables is not a well-known matter, thus, we should explain what it means. Consider for example a small neighbourhood $U\subset C^{2}$ of a point on $L_{1}^{\left(1\right)}$, which is not a crossing point of two such lines. Note that the complex variable

$$z_{1}=x_{1}+ix_{2}-(X_{1}^{\left(1\right)}+iX_{2}^{\left(1\right)})$$

is a coordinate transversal to $L_{1}^{\left(1\right)}$. The 2-line $L_{1}^{\left(1\right)}$ corresponds to *z*_1_ = 0. The complex variable

$$z_{2}=x_{1}-ix_{2}$$

is then a coordinate tangential to $L_{1}^{\left(1\right)}$.

Let *A* be some point in *U*. Consider a path (oriented contour) *σ* in *U* starting and ending at *A*, and having no intersections with $L_{1}^{\left(1\right)}$. Such a contour, called a *bypass* of $L_{1}^{\left(1\right)}$, can be projected onto the variable *z*_1_. Denote this projection by *σ*′. One can continue *u*_c_ along *σ* and obtain the branch *u*_c_(*A* ;*σ*). Branches can be indexed by an integer *p*, which is the winding number of *σ*′ about zero. If for some *σ*′ having winding number *p*

$$u_{\text{c}}(A;\sigma )=u_{\text{c}}(A)$$

for any such continuation (here we consider the smallest possible *p* having this property), then the branch line $L_{1}^{\left(1\right)}$ has order of branching equal to *p*. If there is no such *p*, the branching is said to be logarithmic.

Thus, generally speaking, the branching of a function of several complex variables is similar to that of a single variable, and it is convenient to study this branching using a transversal complex coordinate. To provide the existence of such a transversal variable, the branch set should be a set (a complex manifold) of complex codimension 1.

For *j*, *k* ∈ {1, …*N*}, the 2-lines $L_{1}^{\left(j\right)}$ and $L_{2}^{\left(k\right)}$ intersect at a single point. For example, if *j* = *k*, this point is *P*_*j*_, while for *j* ≠ *k*, this intersection point does not belong to $R^{2}$. The branching of *u*_c_ near each crossing point has a property that does not occur in the one-dimensional complex case: the bypasses about $L_{1}^{\left(j\right)}$ and $L_{2}^{\left(k\right)}$ commute.

Let us prove this in the case *j* = *k* by considering a small neighbourhood $U\subset C^{2}$ of the point $P_{j}=L_{1}^{\left(j\right)}\cap L_{2}^{\left(j\right)}$. The case *j* ≠ *k* is similar. Introduce the local coordinates

$$z_{1}=x_{1}+ix_{2}-(X_{1}^{\left(j\right)}+iX_{2}^{\left(j\right)}),\;z_{2}=x_{1}-ix_{2}-(X_{1}^{\left(j\right)}-iX_{2}^{\left(j\right)}),$$

which are transversal variables to $L_{1}^{\left(j\right)}$ and $L_{2}^{\left(j\right)}$, respectively. Take a point $A\in U\setminus (L_{1}^{\left(j\right)}\cup L_{2}^{\left(j\right)})$ and a path *σ* in $U\setminus (L_{1}^{\left(j\right)}\cup L_{2}^{\left(j\right)})$ starting and ending at *A*. Consider the projections *σ*_1_ and *σ*_2_ of *σ* onto the complex planes of *z*_1_ and *z*_2_, respectively.

Assume that the path *σ* is parametrized by a real parameter *τ* ∈ [0, 1], i.e.

$$\sigma :\;(x_{1}(\tau ),x_{2}(\tau )),$$

or, in the new coordinates,

$$\sigma :\;(z_{1}(\tau ),z_{2}(\tau )).$$

The path *σ* can be deformed homotopically into a path $\sigma^{*}$ defined by

$$\sigma^{*}:\;(\epsilon z_{1}(\tau )/|z_{1}(\tau )|,\epsilon z_{2}(\tau )/|z_{2}(\tau )|),$$

for some small parameter *ϵ*. The projection of $\sigma^{*}$ onto *z*_1_ (resp. *z*_2_) is a small circle $\sigma_{1}^{*}$ (resp. $\sigma_{2}^{*}$) of radius *ϵ* turning (possibly many times) around the origin. Therefore, $\sigma^{*}$ lies on a torus (product of two circles), for which $\sigma_{1}^{*}$ and $\sigma_{2}^{*}$ are strictly longitudinal and latitudinal paths, respectively. Thus these loops commute (this comes from the fact that the fundamental group of the torus is Abelian). Therefore, the path *σ* can be homotopically deformed into the concatenations *σ* = *σ*_1_*σ*_2_ = *σ*_2_*σ*_1_, where the path *σ*_1_ occurs for fixed *z*_2_, and *σ*_2_ occurs for fixed *z*_1_.

## 4. Integral representation of the analytical continuation

Here we present the technique for analytical continuation of the wave field using Green’s third identity as in e.g. [12,15].

Let $U\subset (S\setminus \cup_{j}P_{j})$ be a small neighbourhood of a point *A*_0_ ∈ *S* such that $\psi (A_{0})=(x_{1},x_{2})\in R^{2}$, where *ψ* is the natural projection of *S* to $R^{2}$. In what follows, in an abuse of notation, we may sometimes identify *A*_0_ and *ψ*(*A*_0_) when no ambiguity can arise. Let the contour *γ* be the boundary of *U* oriented anticlockwise with unit external normal vector ***n***. Write Green’s third identity for *A*_0_ ∈ *U*:

4.1 $$u(A_{0})=\int_{\gamma }\left[\frac{\partial G}{\partial n^{\prime }}(\text{r},{\text{r}}^{\prime })u({\text{r}}^{\prime })-\frac{\partial u}{\partial n^{\prime }}({\text{r}}^{\prime })G(\text{r},{\text{r}}^{\prime })\right]\text{d}l^{\prime },$$

where **r**′ = (*x*_1_′, *x*_2_′) is a position vector along *γ*, **r** = (*x*_1_, *x*_2_) points to *A*_0_, ∂/∂*n*′ corresponds to the normal derivative associated to the unit external normal vector, and

4.2 $$G(\text{r},{\text{r}}^{\prime })=-\frac{i}{4}H_{0}^{\left(1\right)}(k\;r(\text{r},{\text{r}}^{\prime }))\;\text{with}\;r(\text{r},{\text{r}}^{\prime })=\sqrt{{\left(x_{1}-x_{1}^{\prime }\right)}^{2}+{\left(x_{2}-x_{2}^{\prime }\right)}^{2}},$$

where $H_{0}^{\left(1\right)}$ is the zeroth-order Hankel function of the first kind. Note that the point *A*_0_ is the only singularity of the integrand of (4.1) in the real (*x*_1_′, *x*_2_′) plane.

The orientation of *γ* plays no role in (4.1), however we can use the orientation of contours to set the direction of the normal vector. Namely, let the normal vector point *to the right* from an oriented contour.

The formula (4.1) can be used to continue *u*(*x*_1_, *x*_2_) to *u*_c_(*x*_1_, *x*_2_) in a small domain of $C^{2}$. Namely let $A\equiv \text{r}=(x_{1},x_{2})\in C^{2}$ be complex, while (*x*_1_′, *x*_2_′) remains real and belongs to *γ*. By small domain we mean that we choose *A* = (*x*_1_, *x*_2_) to be close enough to *A*_0_ so that if we consider a straight path between *A*_0_ and *A*, then, for any $r^{\prime }\in \gamma$, this path does not intersect any of the complex singularities of the Green’s function *G*(**r**, **r′**) seen as a function of (*x*_1_, *x*_2_).

Hence, if (*x*_1_, *x*_2_) is close to *A*_0_, the Green’s function *G*(**r**, **r′**) remains regular for each **r′** ∈ *γ*. Moreover, being considered as a function of **r**, the Green’s function *G*(**r**, **r**′) obeys the Cauchy–Riemann conditions (3.1) and the complex Helmholtz equation (3.2) provided **r** is a regular point for certain fixed **r**′. Thus, for a small complex neighbourhood of *A*_0_ the formula (4.1) provides a function obeying all restrictions (listed in §3) imposed on *u*_c_(*A*).

We should note that the continuation *u*_c_ of *u* in a small complex neighbourhood of *A*_0_ is unique and is provided by letting **r** become a complex vector in (4.1). The proof is given in appendix B and its structure is as follows. We start by deriving a complexified Green’s formula obeyed by *u*_c_ (or by any analytical solution of (3.2)). Then, by Stokes’ theorem, the integration contour for this formula for *A* belonging to some small complex neighbourhood of *A*_0_ can be taken to coincide with *γ*. In this case, the complexified Green’s formula for *u*_c_ coincides with (4.1).

The procedure of analytical continuation of *u* using (4.1) fails when *G*(**r**, **r**′) becomes singular for some **r**′. Let us develop a simple graphical tool to explore the singularities of *G*(**r**, **r**′) for complex **r** ≡ *A*. The function (4.2) is singular when

4.3 $${\left(x_{1}-x_{1}^{\prime }\right)}^{2}+{\left(x_{2}-x_{2}^{\prime }\right)}^{2}=0,$$

i.e. when *A* ≡ (*x*_1_, *x*_2_) and *A*′ ≡ (*x*_1_′, *x*_2_′) both belong to some characteristic of (3.2). Let us fix the complex point *A* and find the real points *A*′ ≡ (*x*_1_′, *x*_2_′) such that (4.3) is valid. Obviously, *A*′ can have two values

4.4 $$A_{1}=(\text{Re}[x_{1}]-\text{Im}[x_{2}],\text{Im}[x_{1}]+\text{Re}[x_{2}])$$

and

4.5 $$A_{2}=(\text{Re}[x_{1}]+\text{Im}[x_{2}],\text{Re}[x_{2}]-\text{Im}[x_{1}]).$$

These two points coincide when *A* is a real point. We will call *A*_1_ and *A*_2_ the *first and the second real points associated with *A** and will sometimes use the notation *A*_1_(*A*) and *A*_2_(*A*) to emphasize the link between *A*_1,2_ and *A*. Both points *A*_1_ and *A*_2_ belong to $R^{2}$, but by the slight abuse of notation mentioned above we can also consider them to be on *S*.

The Green’s function *G*(**r**, **r**′) is singular at some point of the integral contour *γ* in (4.1) if *A*_1_(*A*) ∈ *γ* or *A*_2_(*A*) ∈ *γ*, where **r** points to *A*.

Consider the analytical continuation along a simple path *σ* as a continuous process. Let *σ* be parametrized by a real parameter *τ* ∈ [0, 1], i.e. let each point on the contour *σ* be denoted by *A*(*τ*). Let *A*(0) = *A*_0_ ∈ *S* be the starting real point, and let *A*(1) be the ending complex point. For each point *A*(*τ*) find the associated real points *A*_1,2_ (*τ*) ≡ *A*_1,2_ (*A*(*τ*)). The position of these points depends continuously on *τ*.

We are now well-equipped to formulate the first theorem of analytical continuation.

**Theorem 4.1.**

*Let* $A_{0}\in S\setminus \cup_{j}P_{j}$ *and let* $A\in C^{2}$ *be within a small neighbourhood of A*_0_. *Let σ be a simple path from A*_0_ *to A parametrized by τ* ∈ [0, 1] *as above. Let Γ*(*τ*) ⊂ *S be a continuous set of closed smooth oriented contours (i.e. a homotopical deformation of a contour) such that*

— *Γ*(0) = *γ*;
— *for each τ* ∈ [0, 1] $A_{1}(\tau )\notin \psi (\Gamma (\tau ))$, $A_{2}(\tau )\notin \psi (\Gamma (\tau ))$.

*Then the formula*

4.6 $$u_{\text{c}}(A(\tau )+\delta \text{r})=\int_{\Gamma (\tau )}\left[\frac{\partial G}{\partial n^{\prime }}(\text{r},{\text{r}}^{\prime })u({\text{r}}^{\prime })-\frac{\partial u}{\partial n^{\prime }}({\text{r}}^{\prime })G(\text{r},{\text{r}}^{\prime })\right]dl^{\prime },$$

*defines an analytical continuation u*_c_ *of u in a narrow neighbourhood of σ*. *δ***r** *is an arbitrary small-enough complex radius vector, while the radius-vector* **r** *points to A*(*τ*) + *δ***r**. *Here, by arbitrary small, we mean that* δr *should be small enough so that the two associated real points* $A_{1,2}(A(\tau )+\delta r)$ *remain within the interior of Γ*(*τ*).

**Proof.**

We present a sketch of the proof on the ‘physical level of rigour’. Let the contour *Γ*(*τ*) be changing incrementally, i.e. consider the contours *Γ*(*τ*_*n*_), where 0 = *τ*_0_ < *τ*_1_ < *τ*_2_… < *τ*_*K*_ = 1 is a dense grid on the segment [0, 1]. Each fixed contour *Γ*(*τ*_*n*_) provides an analytic function *u*_c_ in a small neighbourhood of the point *A*(*τ*_*n*_). The grid is dense enough to ensure that such neighbourhoods are overlapping. Moreover, for any point belonging to an intersection of neighbourhoods of *A*(*τ*_*n*_) and *A*(*τ*_*n*+1_) one can deform the contour *Γ*(*τ*_*n*_) into *Γ*(*τ*_*n*+1_) homotopically without changing the value of the integral, and hence without changing the value of *u*_c_. ▪

Note that, formally, the expression (4.6) defines the field *u*_c_ ambiguously. The values of *u* and ∂_*n*′_ *u* on *S* are found in a unique way, but the values of *G* and ∂_*n*′_ *G* should be clarified. Namely, according to (4.2), the value depends on the branch of the square root and of the Hankel function (having a logarithmic branch point at zero).

For *τ* = 0, let *G* be defined on *γ* = *Γ*(0) in the ‘usual’ way: the square root is real positive, and the values of $H_{0}^{\left(1\right)}(\cdot )$ are belonging to the main branch of this function. Then, as *τ* changes continuously from *τ* = 0 to 1, define the values of *G* and ∂_*n*′_ *G* by continuity. Since the (moving) contour *Γ*(*τ*) does not hit the (moving) singular points *A*_1_ (*τ*), *A*_2_ (*τ*), the branch of *G* is defined consistently.

The last theorem in this section extends the local result of theorem 4.1 to a global result.

**Theorem 4.2.**

*Let B be a point of* $C^{2}\setminus (T\cup R^{2})$, *where T is the union of all the 2-lines* $L_{1,2}^{\left(j\right)}$. *Let A*_0_ *be a point belonging to* $S\setminus (\cup_{j}P_{j})$ *and let σ be a smooth path connecting A*_0_ *with B*, *such that*

$$(\sigma \setminus A_{0})\cap (T\cup R^{2})=\emptyset .$$

*Then there exists a family of contours Γ*(*τ*) *associated with σ and obeying the conditions of* theorem 4.1.

We omit the proof of this theorem. It is almost obvious, but not easy to formalize. One should consider the process of changing *τ* from 0 to 1, and ‘pushing’ the already built contour *Γ*(*τ*), which is considered to be movable, by the moving points *A*_1_ (*τ*) and *A*_2_ (*τ*) (or, to be more precise, by small discs centred at *A*_1_ (*τ*) and *A*_2_ (*τ*)).

Some issues may occur with such deformation. For example, the contour may become pinched between *A*_1,2_(*τ*) and *P*_*j*_ for some *j*, or between *A*_1_(*τ*) and *A*_2_(*τ*). The condition

$$(\sigma \setminus A_{0})\cap T=\emptyset$$

guarantees that the points *A*_1_(*τ*), *A*_2_ (*τ*) do not pass through *P*_*j*_, and thus the contour *Γ*(*τ*) cannot be pinched between *A*_1,2_ and *P*_*j*_. The condition

$$(\sigma \setminus A_{0})\cap R^{2}=\emptyset$$

guarantees that the contour *Γ*(*τ*) cannot be pinched between *A*_1_ (*τ*) and *A*_2_ (*τ*).

Theorems 4.1 and 4.2 demonstrate that *u*_c_ can be expressed almost everywhere as an integral (4.6) containing *u*(*x*_1_, *x*_2_) defined on the real surface *S*, and some known kernel *G*. The continuation *u*_c_ has a more complicated structure than *u* on *S*. This is explained by the fact that the set of closed contours on *S* has a more complicated structure than *S* itself. This will be investigated in the next section.

## 5. Analysis of the analytical continuation branching

We will now study the branching of *u*_c_ as follows. Let us consider a starting point $A_{0}\in (S\setminus \cup_{j}P_{j}$), an ending point *B* located near a set on which we study the branching (i.e. near $R^{2}$ or $L_{1,2}^{\left(j\right)}$), and a simple path *σ* connecting *A*_0_ to *B* satisfying

$$(\sigma \setminus A_{0})\cap (T\cup R^{2})=\emptyset ,$$

i.e. *σ* is such that the condition of theorem 4.2 is valid. According to the theorems 4.1 and 4.2, one can continue *u*_c_ from *A*_0_ to *B* along *σ*. Let the result of this continuation be denoted by *u*_c_(*B*). Let *σ*′ be a local path starting and ending at *B* and encircling a corresponding fragment of the branch set. For example, one can introduce a local transversal variable near the branch set and build a contour *σ*′ in the plane of this variable encircling zero for a single time in the positive direction. One can consider a continuation of *u*_c_(*B*) along *σ*′ and obtain a branch of *u*_c_ that is denoted by *u*_c_(*B* ;*σ*′). This is a continuation of *u*_c_ from *A*_0_ along the concatenation *σσ*′ of the contours *σ* and *σ*′. Let the parameter *τ* parametrize the contour *σσ*′; *τ* = 0 correspond to *A*_0_; *τ* = 1/2 correspond to the end of *σ*; and *τ* = 1 correspond to the end of *σσ*′. Consistently with this parametrization, the contour of integration used to obtain *u*_c_(*B*) and *u*_c_(*B* ;*σ*′) will be denoted by *Γ*(1/2) and *Γ*(1), respectively.

If *u*_c_(*B* ;*σ*′) ≡ *u*_c_(*B*), then the contour *σ*′ yields no branching. If *u*_c_(*B* ;(*σ*′)^*p*^) ≡ *u*_c_(*B*), for some integer *p* > 1, then the branching has order *p* (the smallest strictly positive integer with such property). Here *u*_c_(*B* ;(*σ*′)^*p*^) is the result of continuation along the concatenation *σσ*′…*σ*′, where *σ*′ is taken *p* times. The two following theorems establish the type of branching of *u*_c_.

**Theorem 5.1.**

*The points of the real plane* $R^{2}$ *other than P*_*j*_ *do not belong to the branch set of u*_c_.

**Proof.**

Note that $R^{2}$ is not an analytical set, so the concept of a transversal variable is not fully applicable to it (and of course this is the reason of non-branching at it). Anyway, the scheme sketched above can be applied to this case. Let *B* be close to $R^{2}$ but not close to any of the *P*_*j*_. Consider a local path *σ*′ starting and ending at *B* and encircling $R^{2}$. Consider the motion of *A*_1_(*A*) and *A*_2_(*A*) as the point *A* travels along *σ*′. To be consistent with the parametrization, we will refer to these points as *A*_1,2_(*τ*) for *τ* ∈ [1/2, 1]. These points are close to each other and close to *B* since *B* is close to $R^{2}$. Depending on the particular contour *σ*′, it may happen that during this motion *A*_2_ encircles *A*_1_ a single time, several times or not at all. Note that *A*_1_(1/2) = *A*_1_ (1) = *A*_1_(*B*) and *A*_2_(1/2) = *A*_2_ (1) = *A*_2_(*B*).

One can show that if *A*_2_ does not encircle *A*_1_ then the contour *Γ*(1) is homotopic to *Γ*(1/2), and thus *u*_c_(*B* ;*σ*′) = *u*_c_(*B*). This can be seen by considering a small disc around *A*_2_, and thinking of *Γ*(*τ*) as an elastic band that remains glued to that disc once in contact and deforms accordingly, without intersecting *A*_1_, as *A*_2_ moves. Since *A*_2_ does not encircle *A*_1_, then neither does the resulting contour. Hence, once *A*_2_ is back to its original position, the contour can easily be deformed back to *Γ*(1/2).

Let *A*_2_ encircle *A*_1_ a single time. The case of several times follows from this case in a clear way. To consider this case, we follow the principles of computation of ramified integrals that can be found for example in [12,14,18]. Namely, it is known that the integral changes locally during a local bypass. The fragments of the contour *Γ*(1/2) that are located far from the points *A*_1_(1/2) and *A*_2_(1/2) are not affected by the bypass *σ*′. The fragments that are close to these points but do not pass between these points are not affected either. The only parts that are affected are the parts of *Γ*(1/2) passing between the points *A*_1_(1/2) and *A*_2_ (1/2).

**Figure 3.**
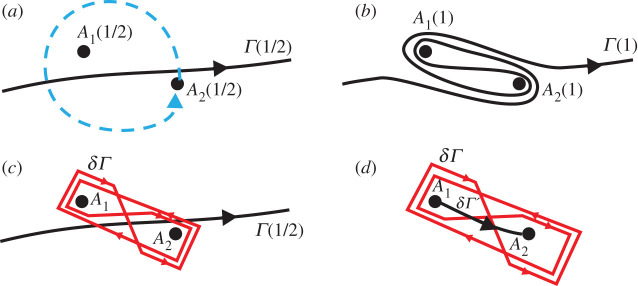
(*a*) Initial configuration, (*b*) continuous deformed contour obtained after *A*_2_ has performed a full circle, (*c*) the deformed contour is written as a sum of the initial contour and a double-eight contour and (*d*) illustration of the connection contour *δΓ* (Online version in colour.)

In figure 3 we demonstrate the deformation of a fragment of *Γ*(1/2) (figure 3*a* into a corresponding fragment of *Γ*(1) (figure 3*b*). One can see from figure 3*c* that the fragment of *Γ*(1) can be written as the sum of the initial fragment of *Γ*(1/2) and an additional contour *δΓ*. The contour *δΓ* is a ‘double-eight’ contour having the following property: it bypasses each of the points *A*_1_(1/2) and *A*_2_(1/2) zero times totally (one time in the positive and one time in the negative direction). We say that this double-eight contour is based on the points *A*_1_(1/2) and *A*_2_(1/2). Such a contour is also known as a Pochhammer contour.

Since there may be several parts of *Γ*(1/2) passing between *A*_1_(1/2) and *A*_2_(1/2), the contour *Γ*(1) can be written as a sum of *Γ*(1/2) and several double-eight contours based on the points *A*_1_(1/2) and *A*_2_(1/2). These contours, possibly, drawn on different sheets of *S*.

Let us show that for a double-eight contour *δΓ* based on *A*_1_(1/2) and *A*_2_(1/2), we have

5.1 $$\int_{\delta \Gamma }\left[\frac{\partial G}{\partial n^{\prime }}(\text{r},{\text{r}}^{\prime })u({\text{r}}^{\prime })-\frac{\partial u}{\partial n^{\prime }}({\text{r}}^{\prime })G(\text{r},{\text{r}}^{\prime })\right]\text{d}l^{\prime }=0.$$

This will yield that *u*_c_(*B*;*σ*′) = *u*_c_(*B*) and that there is no branching on $R^{2}$. In order to do so, connect the points *A*_1_(1/2) and *A*_2_(1/2) with an oriented contour *δΓ*′ (see figure 3*d*, *δΓ*′ is shown by a black line). Squeeze the contour *δΓ* in such a way that it goes four times along *δΓ*′.

We now claim that it is not necessary to account for the integral in the close vicinity of *A*_1_(1/2) or *A*_2_(1/2). Indeed, using (4.2), (4.4) and (4.5), one can show that the kernel *G* has two logarithmic branch points at *A*_1_(1/2) and *A*_2_(1/2), and hence, in principle, the contour *δΓ* needs to be considered on the Sommerfeld surface of *G*. This surface has infinitely many sheets, and can be constructed by considering a straight cut between *A*_1_(1/2) and *A*_2_(1/2) and suitable ‘gluing’. What is important is that locally, around *A*_1_(1/2) and *A*_2_(1/2), *G* behaves like a complex logarithm. Hence, locally, the difference in *G* between two adjacent sheets is constant. Therefore, when considering the part of *δΓ* in the vicinity of *A*_1_(1/2) and *A*_2_(1/2), two loops going in opposite directions, the overall contribution of the integral tends to zero as these loops ‘shrink’ to *A*_1,2_(1/2). This is due to the fact that, *u* being single valued on *δΓ*, the logarithmic singularities of *G* cancel out. Thus, a consideration of the integral near *A*_1_(1/2) and *A*_2_(1/2) is not necessary, and it is possible to reduce *δΓ* to four copies of *δΓ*′.

As explained in detail in appendix C, according to the formulae (4.4)–(4.5) linking the complex variables (*x*_1_, *x*_2_) with the real coordinates of the points *A*_1_, *A*_2_, and to formulae (4.2), a bypass about *A*_1_ in the positive direction (in the real plane) leads to the following change of the argument of $H_{0}^{\left(1\right)}(z)$: *z* → *e*^*iπ*^ *z*. Similarly, a bypass about *A*_2_ in the positive direction changes the argument of the Hankel function as *z* → *e*^−*iπ*^ *z*.

As a result, one can rewrite the integral of (5.1) as

5.2 $$\int_{\delta \Gamma }\left[\frac{\partial G}{\partial n^{\prime }}(\text{r},{\text{r}}^{\prime })u({\text{r}}^{\prime })-\frac{\partial u}{\partial n^{\prime }}({\text{r}}^{\prime })G(\text{r},{\text{r}}^{\prime })\right]\text{d}l^{\prime }=\int_{\delta \Gamma^{\prime }}\left[\frac{\partial G^{\prime }}{\partial n^{\prime }}(\text{r},{\text{r}}^{\prime })u({\text{r}}^{\prime })-\frac{\partial u}{\partial n^{\prime }}({\text{r}}^{\prime })G^{\prime }(\text{r},{\text{r}}^{\prime })\right]\text{d}l^{\prime },$$

where

5.3 $$G^{\prime }(\text{r},{\text{r}}^{\prime })=-\frac{i}{4}(-H_{0}^{\left(1\right)}(e^{-i\pi }k\;r(\text{r},{\text{r}}^{\prime }))+2H_{0}^{\left(1\right)}(k\;r(\text{r},{\text{r}}^{\prime }))-H_{0}^{\left(1\right)}(e^{i\pi }k\;r(\text{r},{\text{r}}^{\prime }))).$$

The notation $H_{0}^{\left(1\right)}(e^{i\pi }z)$ means the value of $H_{0}^{\left(1\right)}(\cdot )$ obtained as the result of continuous rotation of the argument *z* about the origin for the angle *π* in the positive direction.

Apply the formula (see e.g. [19], sec. 1.33, eqns (202)–(203)) well-known from the theory of Bessel functions:

5.4 $$-H_{0}^{\left(1\right)}(e^{-i\pi }z)+2H_{0}^{\left(1\right)}(z)-H_{0}^{\left(1\right)}(e^{i\pi }z)=0,$$

to conclude that the expression on the right-hand side of (5.2) is equal to zero and conclude the proof. Here and below, the formula (5.4) seems to play a fundamental role in the process of analytical continuation. ▪

**Figure 4.**
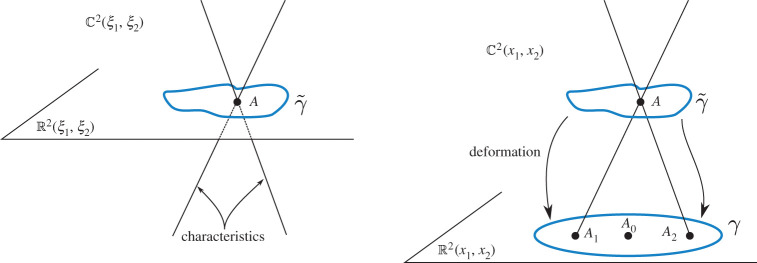
Diagrammatic Illustration of the contours $\tilde{\gamma }$ and *γ* and how one is deformed to the other. (Online version in colour.)

Note also that every branch of *u*_*c*_ is continuous at any point $A_{0}\in R^{2}\setminus \cup_{j}P_{j}$, thus these points are regular points of any branch of *u*_c_. This can be seen using the complexified Green’s theorem of appendix B. Indeed, using the same idea of variable translation, it can be shown that for a point $A\in C^{2}\setminus (T\cup R^{2})$ in a close complex neighbourhood of *A*_0_ and a given branch *u*_*c*_(*A*), we can write $u_{c}(A)=\int_{\tilde{\gamma }}(u_{c}\nabla_{C}G-G\nabla_{C}u_{c})$ for some small contour $\tilde{\gamma }$ surrounding *A* in $C^{2}$. Since the differential form $u_{c}\nabla_{C}G-G\nabla_{C}u_{c}$ is closed (see appendix B), Stokes’ theorem allows us to deform $\tilde{\gamma }$ to a contour *γ* in $R^{2}$ surrounding *A*_0_ (see figure 4) without changing the value of the integral. *A* is chosen close enough to *A*_0_ so that we can let *A* → *A*_0_ along a simple small straight path without any singularities of the integrand hitting the contour *γ*, showing, in doing so, the continuity of *u*_*c*_ at *A*_0_.

**Theorem 5.2.**

*Let* $P_{j}\in R^{2}$ *be a branch point of order p* (*the definition of the order of the branch point is clarified below*). *Then both* $L_{1}^{\left(j\right)}$ *and* $L_{2}^{\left(j\right)}$ *are branch 2-lines of u*_c_ *of order p*.

**Proof.**

First, let us define what it exactly means for $P_{j}\in R^{2}$ to be a branch point of *S* of order *p*. The situation is simple if *ψ*^−1^ (*P*_*j*_) is a single point. Then *p* is the order of branching of *S* at *ψ*^−1^(*P*_*j*_). If *ψ*^−1^ (*P*_*j*_) is a set of several points, then *p* is the least common multiple of orders of all points *ψ*^−1^ (*P*_*j*_). A bypass encircling *P*_*j*_ *p* times in the positive direction returns each point located closely to *P*_*j*_ to itself.

Consider a point $B\in C^{2}\setminus T$ that is close to $L_{1}^{\left(j\right)}$. Let the loop *σ*′ bypass $L_{1}^{\left(j\right)}$ once in the positive direction (i.e. its projection onto the local transversal coordinate

$$z_{1}=x_{1}+ix_{2}-(X_{1}^{\left(j\right)}+iX_{2}^{\left(j\right)})$$

bypasses zero once in the positive direction). Let *σ*′ be also such that it does not bypass any other 2-line of *T*. As before, parametrize *σ*′ by *τ* ∈ [1/2, 1] and denote by *Γ*(*τ*) the associated integration contours used for the analytical continuation along *σ*′ . Then *A*_1_(*τ*) bypasses *P*_*j*_ once in the positive direction, and *A*_2_(*τ*) does not bypass any branch point.

The fragments of *Γ*(1/2) that are far from *A*_1_ (1/2) or not passing between *A*_1_ (1/2) and *P*_*j*_ are not affected by *σ*′. For each fragment of *Γ*(1/2) passing between *A*_1_ (1/2) and *P*_*j*_, the double-eight contour *δΓ* is added to obtain a fragment of *Γ*(1). In this case, the double-eight contour is based on the points *A*_1_ (1/2) and *P*_*j*_. The graphical proof is similar to that in figure 3.

**Figure 5.**
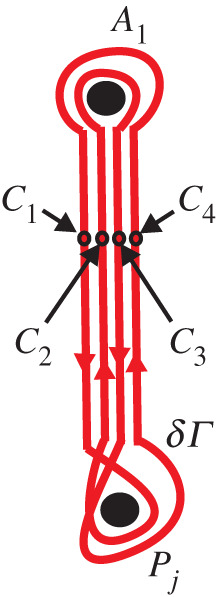
Illustration of *δΓ* and the points of application of formula (5.4) to *δΓ*. (Online version in colour.)

Let us now show that the order of branching at $L_{1}^{\left(j\right)}$ is equal to *p*. Consider a fragment of *Γ*(1/2) passing between *A*_1_ and *P*_*j*_. A single bypass along *σ*′ changes (locally)

$$\Gamma (1/2)\overset{\sigma^{\prime }}{⟶}\Gamma (1/2)+\delta \Gamma ,$$

where *δΓ* is illustrated in figure 5. Upon performing a second bypass *σ*′, we hence get

$$\Gamma (1/2)+\delta \Gamma \overset{\sigma^{\prime }}{⟶}\Gamma (1/2)+\delta \Gamma +\delta \Gamma^{\left(1\right)},$$

where *δΓ*^(1)^ is the double-eight contour based on the points *A*_1_ and *P*_*j*_, and obtained from *δΓ* by rotating *A*_1_ about *P*_*j*_ once in the positive direction.

The projection *ψ*(*δΓ*^(1)^) coincides with *ψ*(*δΓ*), however, the new contour passes along other sheets of *S*, and the branch of $H_{0}^{\left(1\right)}$ is different on it. Finally, after *p* rotations one gets the integration contour *Γ*(1/2) + *δΓ* + *δΓ*^(1)^ + · · · + *δΓ*^(*p*−1)^. In order to show that the branching of $L_{1}^{\left(j\right)}$ is of order *p*, we need to show that

5.5 $$\int_{\delta \Gamma +\delta \Gamma^{\left(1\right)}+\cdots +\delta \Gamma^{\left(p-1\right)}}\left[\frac{\partial G}{\partial n^{\prime }}(\text{r},{\text{r}}^{\prime })u({\text{r}}^{\prime })-\frac{\partial u}{\partial n^{\prime }}({\text{r}}^{\prime })G(\text{r},{\text{r}}^{\prime })\right]\text{d}l^{\prime }=0,$$

implying that *u*_c_(*B* ;(*σ*′)^*p*^) = *u*_c_(*B*), and that the branching has order *p*. Algebraically, in terms of homology classes, this can be written as

5.6 $$\delta \Gamma +\delta \Gamma^{\left(1\right)}+\cdots +\delta \Gamma^{\left(p-1\right)}=0.$$

We will show this by decomposing *δΓ* and all its subsequent ‘copies’ into three main parts: (i) the two circles around *P*_*j*_; (ii) the two circles around *A*_1_; and (iii) the four straight lines between *A*_1_ and *P*_*j*_, and showing that the contribution of each of these three parts to the integral (5.5) is indeed zero.

(i) By Meixner conditions, *u* and ∂_*n*_′*u* are integrable in the close vicinity of *P*_*j*_, where *G*(**r**, · ) is well behaved. Hence we can ‘shrink’ the circles to *P*_*j*_, and the integrals over the two circles become zero and do not contribute to (5.5).
(ii) For the circles around *A*_1_, the argument is slightly more subtle. Let (*s*_1_, …, *s*_*p*_) be the sheets of *S* accessible by turning around *P*_*j*_. Whatever sheet *u* is on the initial outer circle, after *p* rotations, it will have ‘visited’ all sheets *s*_1_, …, *s*_*p*_ once. The same is true for the inner circle. Hence, the overall contribution of the circles to (5.5) can be written as
  5.7 $$\begin{array}{rl} & \int_{A_{1}}\left\{[\frac{\partial G}{\partial n^{\prime }}(\text{r},{\text{r}}^{\prime };h_{1})u({\text{r}}^{\prime };s_{1})-\frac{\partial u}{\partial n^{\prime }}({\text{r}}^{\prime };s_{1})G(\text{r},{\text{r}}^{\prime };h_{1})\right]\\ & +\cdots +\left[\frac{\partial G}{\partial n^{\prime }}(\text{r},{\text{r}}^{\prime };h_{p})u({\text{r}}^{\prime };s_{p})-\frac{\partial u}{\partial n^{\prime }}({\text{r}}^{\prime };s_{p})G(\text{r},{\text{r}}^{\prime };h_{p})\right]\}\text{d}l^{\prime }\\ - & \int_{A_{1}}\left\{[\frac{\partial G}{\partial n^{\prime }}(\text{r},{\text{r}}^{\prime };h_{1}^{\prime })u({\text{r}}^{\prime };s_{1})-\frac{\partial u}{\partial n^{\prime }}({\text{r}}^{\prime };s_{1})G(\text{r},{\text{r}}^{\prime };h_{1}^{\prime })\right]\\ & +\cdots +\left[\frac{\partial G}{\partial n^{\prime }}(\text{r},{\text{r}}^{\prime };h_{p}^{\prime })u({\text{r}}^{\prime };s_{p})-\frac{\partial u}{\partial n^{\prime }}({\text{r}}^{\prime };s_{p})G(\text{r},{\text{r}}^{\prime };h_{p}^{\prime })\right]\}\text{d}l^{\prime }, \end{array}$$
  for some (*h*_1_, …, *h*_*p*_) and (*h*_1_′, …, *h*_*p*_′) corresponding to given sheets of *G*(**r**, · ), where the ; notation in the argument of a function specifies which sheet it is on, and where $\int_{A_{1}}$ specifies integration along a small circle encircling *A*_1_. Now we can group the terms for which *u* lies on the same sheet. For each such pair, we can use the reasoning used below (5.1) in the proof of theorem 5.1 to show that even if *G* may be on a different sheet for each element of the pair, the logarithmic singularities cancel out. Hence one can safely ‘shrink’ the circles to *A*_1_ without leading to any contribution to (5.5).
(iii) Finally, for the straight lines, one can check that due to the identity (5.4), the branch of $H_{0}^{\left(1\right)}$ (and hence of *G*) does not matter. Namely, let *C*_1_, *C*_2_, *C*_3_, *C*_4_ be some points of *δΓ* projected onto a single point $C\in R^{2}$. These points are shown in figure 5, but for clarity they are shown close to each other, not above one another.
  Consider the points *C*_1_ and *C*_4_. They belong to the same sheet of *S*, but the branches of $H_{0}^{\left(1\right)}$ are different at these points. Namely, if the argument of $H_{0}^{\left(1\right)}$ is equal to *z* at *C*_1_, then it is equal to *z* *e*^−*iπ*^ at *C*_4_ (see again appendix C). Since the contours have different directions at *C*_1_ and *C*_4_, the contribution of the two outer lines to the integral over *δΓ* is of the form
  5.8 $$\int_{\delta \Gamma^{\prime }}\left[\frac{\partial G^{\prime }}{\partial n^{\prime }}(\text{r},{\text{r}}^{\prime })u({\text{r}}^{\prime };s)-\frac{\partial u}{\partial n^{\prime }}({\text{r}}^{\prime };s)G^{\prime }(\text{r},{\text{r}}^{\prime })\right]\;\text{d}l^{\prime },$$
  for some *s* ∈ (*s*_1_, …, *s*_*p*_), where $G^{\prime }(\text{r},{\text{r}}^{\prime })=-\frac{i}{4}(H_{0}^{\left(1\right)}(kr(\text{r},{\text{r}}^{\prime }))-H_{0}^{\left(1\right)}(e^{-i\pi }kr(\text{r},{\text{r}}^{\prime }))$ and where *δΓ*′ is a straight line between *A*_1_ and *P*_*j*_.
  Perform a bypass *σ*′. In the course of this bypass, the points *C*_1_ and *C*_4_ encircle *A*_1_ in the positive direction. Thus, the corresponding part of the integral along *δΓ*^(1)^ contains $H_{0}^{\left(1\right)}(ze^{i\pi })-H_{0}^{\left(1\right)}(z)$ and its derivative. However, due to (5.4),
  5.9 $$H_{0}^{\left(1\right)}(z\;e^{i\pi })-H_{0}^{\left(1\right)}(z)=H_{0}^{\left(1\right)}(z)-H_{0}^{\left(1\right)}(z\;e^{-i\pi }),$$
  and hence the value of *G*′ is unaffected by such a bypass. As before, after *p* bypasses, *u* would have ‘visited’ all the sheets (*s*_1_, …, *s*_*p*_) and the overall contribution of the two outer lines to (5.5) can be written
  5.10 $$\int_{\delta \Gamma^{\prime }}\left[\frac{\partial G^{\prime }}{\partial n^{\prime }}(\text{r},{\text{r}}^{\prime })u^{\prime }({\text{r}}^{\prime })-\frac{\partial u^{\prime }}{\partial n^{\prime }}({\text{r}}^{\prime })G^{\prime }(\text{r},{\text{r}}^{\prime })\right]\;\text{d}l^{\prime },$$
  where *u*′( · ) = *u*( · ;*s*_1_) + · · · + *u*( · ;*s*_*p*_).
  The same consideration can be conducted for the points *C*_2_ and *C*_3_ on the inner lines to show an overall contribution equal to minus that of (5.10). Hence the overall contribution of the straight lines to (5.5) is also zero.

We have therefore proved that the equality (5.5) is correct, and hence that $L_{1}^{\left(j\right)}$ is a branch 2-line of order *p*. The case of *σ*′ bypassing $L_{2}^{\left(j\right)}$ can be considered in a similar way. Note that this time, if *σ*′ bypasses $L_{2}^{\left(j\right)}$ once in the positive direction (and no other branch 2-line), then the corresponding *A*_2_ (*τ*) bypasses *P*_*j*_ once in the negative direction, and *A*_1_ (*τ*) does not bypass any branch point. ▪

**Theorem 5.3.**

*Consider two points* $A,B\in (C^{2}\setminus T)$, *and let σ*_1_ *and σ*_2_ *be piecewise-smooth paths in* $\left(C^{2}\setminus T\right)$ *connecting A with B*. *Assume that it is possible to continue homotopically σ*_1_ *to σ*_2_ *in* $\left(C^{2}\setminus T\right)$. *Let u*_c_(*A*) *be some branch of u*_c_ *in some neighbourhood of A*. *Then the branches of u*_c_(*B*) *obtained by continuation of u*_c_(*A*) *along σ*_1_ *and along σ*_2_ *coincide*:

$$u_{\text{c}}(B;\sigma_{1})=u_{\text{c}}(B;\sigma_{2}).$$

This theorem follows naturally from the principle of analytical continuation, which remains valid in two-dimensional complex analysis. In the following theorem, we show that any closed contour in $C^{2}\setminus T$ can be deformed to a concatenation of *canonical building blocks*.

**Theorem 5.4.**

*Let A be a point in* $C^{2}\setminus T$, *and let σ be a closed path in* $C^{2}\setminus T$ *starting and ending at A*. *Then, within* $C^{2}\setminus T$, *σ can be homotopically transformed into a concatenation contour σ*′ *of the form*

5.11 $$\sigma^{\prime }=\sigma_{\alpha_{1}}^{m_{1}}\sigma_{\alpha_{2}}^{m_{2}}\ldots \sigma_{\alpha_{H}}^{m_{H}},$$

*for some positive integer H*. *For h* ∈ {1, …, *H*}, *the powers m*_*h*_ *belong to* Z *and the elementary contours* $\sigma_{\alpha_{h}}$ *are fixed for each α*_*h*_. *The indices α*_*h*_ *are pairs of the type* (ℓ, *j*), *where* ℓ ∈ {1, 2} *and j* ∈ {1, …, *N*}. *These contours can themselves be represented as concatenations*

5.12 $$\sigma_{\alpha_{h}}=\gamma_{\alpha_{h}}\sigma_{\alpha_{h}}^{*}\gamma_{\alpha_{h}}^{-1},$$

*where each contour* $\gamma_{\alpha_{h}}$ *goes from A to some point near* $L_{\ell }^{\left(j\right)}$, *and* $\sigma_{\alpha_{h}}^{*}$ *is a local contour encircling* $L_{\ell }^{\left(j\right)}$ *once. On each contour σ*_1,*j*_ *the value x*_1_ − *ix*_2_ *is constant. On each contour σ*_2,*j*_ *the value x*_1_ + *ix*_2_ *is constant*.

**Figure 6.**
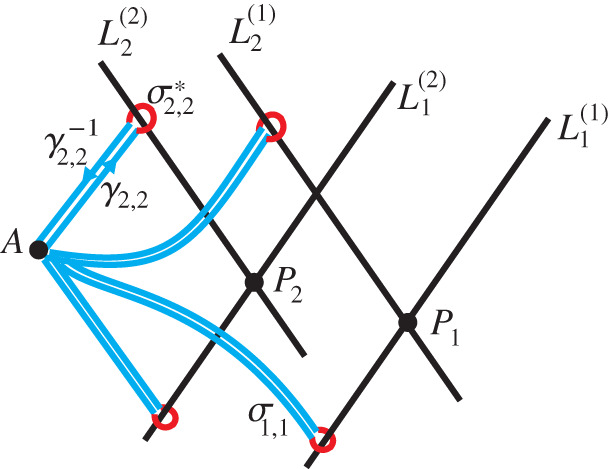
Illustration of some contours $\sigma_{\alpha }$, $\gamma_{\alpha }$ and $\sigma_{\alpha }^{*}$. (Online version in colour.)

Examples of such contours can be found in figure 6.

**Proof.**

Consider a small neighbourhood $U\subset C^{2}\setminus T$ of some point *A*. By the principles of analytic continuation, all possible branches *u*_*c*_(*A*) are obtained by continuation along loops ending and starting at *A* that cover all possible combinations of the homotopy classes of $C^{2}\setminus T$. Each $\sigma_{\alpha }$ represents one of the homotopy classes, and hence by allowing *σ*′ to take the form (5.11), all possible combinations of such classes are covered. Finally since any closed contour *σ* can be written as a combination of homotopy classes, then we can in principle deform any closed contour *σ* to one akin to *σ*′. ▪

Note that the elementary paths *σ*_*α*_ have the following property. Since either *x*_1_ − *i x*_2_ or *x*_1_ + *i x*_2_ is constant on such a path, either *A*_2_ or *A*_1_ remains constant as the path *σ*_*α*_ is passed.

In the next theorem, we formulate the main general result of the paper, which is that there exists a finite basis of elementary functions such that any branch of the analytical continuation *u*_*c*_ is a linear combination of such functions with integer coefficients.

**Theorem 5.5.**

*For a neighbourhood* $U\subset C^{2}\setminus T$ *of a given point A*, *one can find a finite set of basis functions g*_1_ (*A*), …, *g*_*Q*_ (*A*), *A* ∈ *U*, *which are analytical solutions of the complex Helmholtz equation* (3.2) *in U*, *and such that any branch u*_c_(*A* ;*σ*), *can be written as a linear combination*

5.13 $$u_{\text{c}}(A;\sigma )=\sum_{q=1}^{Q}b_{q}(\sigma )g_{q}(A),$$

*where b*_*q*_ (*σ*) *are integer coefficients that are constant with respect to A*. *The dimension of the basis, Q*, *can be defined from the topology of S.*

**Proof.**

According to theorem 4.2, theorem 5.1, theorem 5.2 and the basic principles of analytical continuation, it is enough to prove the statement of the theorem for an arbitrary small neighbourhood *U*.

Indeed, if the theorem is true for such a neighbourhood, then the elements of the basis can be expressed as linear combinations of *Q* linearly independent branches of *u*_c_. The coefficients of the combination are constant, therefore the elements of the basis have singularities only at *T*, and the type of branching is the same as that of *u*_c_. Any other neighbourhood *U*′ can be connected with *U* with a path in $C^{2}\setminus T$, and the formula (5.13) can be continued along this path.

Hence, below, we prove the theorem for a point *A*, for which the mutual location of the points *A*_1,2_ (the real points associated with *A*) and *P*_*j*_ (the branch points on *S*) is convenient in some sense.

**Figure 7.**
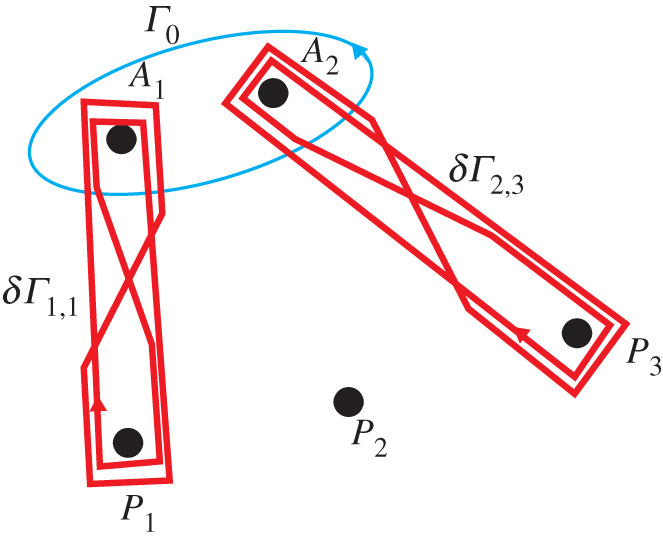
Illustration of some integration contours used to define the basis functions. (Online version in colour.)

Choose the ‘convenient’ neighbourhood *U* as follows. Let us assume that the point *A* ≡ (*x*_1_, *x*_2_) is far enough from the branch points *P*_*j*_, is close to $R^{2}$ but does not belong to $R^{2}$. This results in a configuration akin to that illustrated in figure 7.

Define the basis functions *g*_*q*_ as follows. Let *g*_1_ be defined by the integral (4.6) with the contour *Γ*_0_ shown in figure 7. Such a function *g*_1_ is equal to *u*_c_ obtained by analytical continuation from a small neighbourhood of a point belonging to *S* as per theorem 4.1.

All the other basis functions are constructed as follows. Let *δΓ*_ℓ,*j*_ be a double-eight contour based on the points *A*_ℓ_, ℓ ∈ {1, 2}, and *P*_*j*_, *j* ∈ {1, …, *N*}, as illustrated in figure 7. Consider all preimages *ψ*^−1^(*δΓ*_ℓ,*j*_) on *S*. Denote them by $\delta \Gamma_{\ell ,j}^{\left(1\right)},\ldots ,\delta \Gamma_{\ell ,j}^{\left(M\right)}$, where we remind the reader that *M* is the finite number of sheets of *S*. Some of them are linearly independent. The functions *g*_2_, …, *g*_*Q*_ are the integrals of the form (4.6) taken with all linearly independent contours from the set $\left\{\delta \Gamma_{\ell ,j}^{\left(1\right)},\ldots ,\delta \Gamma_{\ell ,j}^{\left(M\right)}\right\}$.

To see this, let us continue the function *u*_c_ from *A* along some closed path *σ*. Deform the path *σ* into a path *σ*′ as in theorem 5.4. Each building block of *σ*′, *σ*_ℓ,*j*_, can be analysed by the procedure described in the proof of theorem 5.2. A local contour $\sigma_{\ell ,j}^{*}$ produces several local double-eight loops, and the path $\gamma_{\ell ,j}^{-1}$ stretches the loops into those shown in figure 7. ▪

As mentioned in the statement of theorem 5.5, the exact value of the dimension *Q* of the basis depends on the topology of *S* and hence on the specific diffraction problem considered. In the next section we show (among other results) that in the case of the Dirichlet strip problem, we have *Q* = 4.

## 6. The strip problem

Everything so far has been done for a generic scattering problem described in the Introduction. We will here deal with the specific problem of diffraction by a finite strip ( − *a* < *x*_1_ < *a*, *x*_2_ = 0) . The canonical problem of diffraction by a strip has attracted a lot of attention since the beginning of the twentieth century, and various innovative mathematical methods have been designed and implemented to solve it: Schwarzschild’s series [20], Mathieu functions expansion [21], modified Wiener–Hopf technique [22,23], embedding and reduction to ODEs [24,25]. It has important applications, including in aero- and hydro-acoustics, see [26] for example.

The problem can be formulated as follows: find the total field *u* satisfying the Helmholtz equation and Dirichlet boundary conditions (*u* = 0) on the strip, resulting from an incident plane wave. The scattered field (the difference between the total and the incident field) should satisfy the Sommerfeld radiation condition, and the total field should satisfy the Meixner conditions at the edges of the strip.

Here we assume that this physical field *u* is known and we consider the associated Sommerfeld surface *S*. It has two branch points *P*_1_ = (*a*, 0) and *P*_2_ = ( − *a*, 0), so that *N* = 2. They are each of order 2, and the Sommerfeld surface *S* has two sheets (*M* = 2). The surface *S* is shown in figure 1*b*. Let us assume that sheet 1 is the physical sheet, while sheet 2 is its mirror reflection.

We will now apply the general theory developed in the paper in order to unveil the analytical continuation *u*_*c*_.

**Figure 8.**
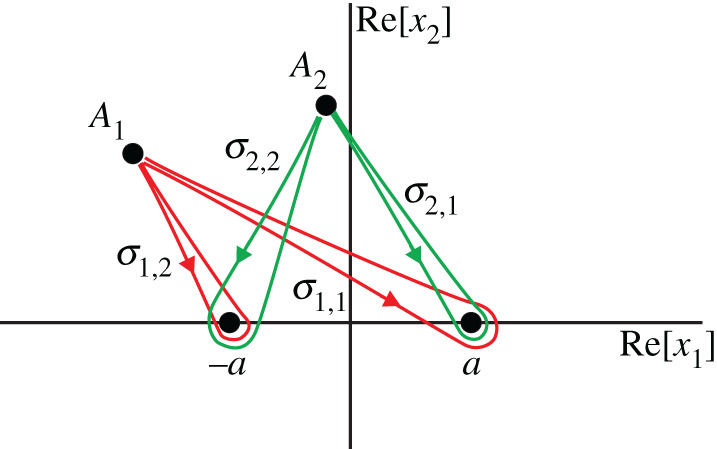
Trajectories of *A*_1,2_ corresponding to the elementary paths *σ*_ℓ,*j*_. (Online version in colour.)

Let $A\equiv (x_{1},x_{2})\in C^{2}\setminus (T\cup R^{2})$ be some point near $R^{2}$. Consider all possible continuations of *u*_c_(*A*) along closed paths. According to theorem 5.4, any such path can be represented as a concatenation of elementary paths *σ*_ℓ,*j*_ for ℓ ∈ {1, 2} and *j* ∈ {1, 2}. The elementary paths can be chosen in a such a way that corresponding trajectories of the points *A*_1_ and *A*_2_ are as shown in figure 8.

As it follows from the considerations in previous sections, each path *σ*_ℓ,*j*_ is an elementary bypass about the branch 2-line $L_{\ell }^{\left(j\right)}$. The 2-lines $L_{1}^{\left(j\right)}$ are bypassed in the positive direction, while the 2-lines $L_{2}^{\left(j\right)}$ are bypassed in the negative direction.

**Figure 9.**
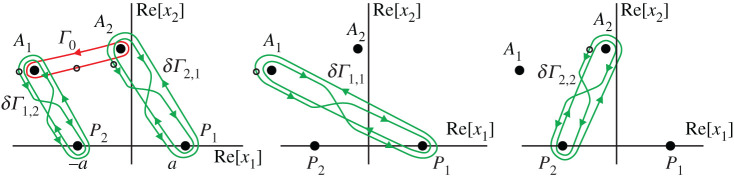
Basis contours for diffraction by a segment. (Online version in colour.)

Now let us choose the basis functions *g*_*j*_ of theorem 5.5. For this, consider the contours *Γ*_0_ and the double-eight loops *δΓ*_ℓ,*j*_ for ℓ, *j* ∈ {1, 2} shown in figure 9. For definiteness, we assume that the points marked by a small black circle on the contours belong to the physical sheet of *S*.

Formally, since the branch points *P*_*j*_ are of order 2, there should exist two double-eight loops for each pair of indexes ℓ, *j*: *δΓ*_ℓ,*j*_ and $\delta \Gamma_{\ell ,j}^{\left(1\right)}$. However, due to the identity (5.6), the loops $\delta \Gamma_{\ell ,j}^{\left(1\right)}$ need not be included in the basis, reducing the number of candidates for the basis functions from nine to five.

Moreover, using contour deformation and cancellation, one can check the following contour identity

6.1 $$\delta \Gamma_{1,1}+\delta \Gamma_{2,1}=\delta \Gamma_{1,2}+\delta \Gamma_{2,2},$$

by showing that each side of (6.1) is equal to 2*Γ*_0_. Therefore, one of the double-eight loops, for example *δΓ*_2,2_, needs not be included in the basis since it depends linearly on the other three contours. This effectively reduces the number of basis functions to four, as required and these basis functions can be explicitly written as

6.2 $$g_{1}(x_{1},x_{2})=\int_{\Gamma_{0}}[\ldots ]\text{d}l^{\prime },\;g_{2}(x_{1},x_{2})=\int_{\delta \Gamma_{1,1}}[\ldots ]\text{d}l^{\prime },$$

6.3 $$g_{3}(x_{1},x_{2})=\int_{\delta \Gamma_{1,2}}[\ldots ]\text{d}l^{\prime },\;g_{4}(x_{1},x_{2})=\int_{\delta \Gamma_{2,1}}[\ldots ]\text{d}l^{\prime },$$

where the […] integrand is the same as in (4.6).

Upon introducing the vector of functions

6.4 $$\text{W}={\left(g_{1},g_{2},g_{3},g_{4}\right)}^{T},$$

the analytic continuation of *u*_c_ is fully described by the following theorem.

**Theorem 6.1.**

*The analytical continuation along each closed fundamental path σ*_ℓ,*j*_ *affects the vector of basis functions as follows*:

6.5 $$\text{W}\overset{\sigma_{\ell ,j}}{⟶}{\text{M}}_{\ell ,j}\text{W},$$

*where the* 4 × 4 *constant matrices M*_ℓ,*j*_ *are given by*

6.6 $${\text{M}}_{1,1}=\left(\begin{array}{cccc} 1 & -1 & 0 & 0\\ 0 & -1 & 0 & 0\\ 0 & -2 & 1 & 0\\ 0 & 0 & 0 & 1 \end{array}\right),\;{\text{M}}_{1,2}=\left(\begin{array}{cccc} 1 & 0 & -1 & 0\\ 0 & 1 & -2 & 0\\ 0 & 0 & -1 & 0\\ 0 & 0 & 0 & 1 \end{array}\right),$$

6.7 $${\text{M}}_{2,1}=\left(\begin{array}{cccc} 1 & 0 & 0 & -1\\ 0 & 1 & 0 & 0\\ 0 & 0 & 1 & 0\\ 0 & 0 & 0 & -1 \end{array}\right),\;{\text{M}}_{2,2}=\left(\begin{array}{cccc} 1 & -1 & 1 & -1\\ 0 & 1 & 0 & 0\\ 0 & 0 & 1 & 0\\ 0 & -2 & 2 & -1 \end{array}\right).$$

The statement of the theorem can be checked directly by studying how the double-eight contours are transformed under the action of each *σ*_ℓ,*j*_, though it is omitted here for brevity.

Nevertheless, the following theorem and its proof can be considered as a check that the matrices given in theorem 6.1 are indeed correct.

**Theorem 6.2.**

*Let j*, *k*, ℓ ∈ {1, 2}. *The following statements are correct*:

(a) *Each branch 2-line* $L_{\ell }^{\left(j\right)}$ *has order 2, i.e*.:
  6.8 $$u_{\text{c}}(A;{\left(\sigma_{\ell ,j}\right)}^{2})=u_{\text{c}}(A).$$
(b) *The bypasses σ*_1,*j*_ *and σ*_2,*k*_ *commute for any pair j*, *k*:
  6.9 $$u_{\text{c}}(A;\sigma_{1,j}\sigma_{2,k})=u_{\text{c}}(A;\sigma_{2,k}\sigma_{1,j}).$$
(c) *The intersecting branch 2-lines* $L_{1}^{\left(j\right)}$ *and* $L_{2}^{\left(k\right)}$ *have the additive crossing property*:
  6.10 $$u_{\text{c}}(A)+u_{\text{c}}(A;\sigma_{1,j}\sigma_{2,k})=u_{\text{c}}(A;\sigma_{1,j})+u_{\text{c}}(A;\sigma_{2,k}).$$

**Proof.**

To prove (a) it is enough to show that for all *j*, ℓ ∈ {1, 2}, we have

$${\text{M}}_{\ell ,j}^{2}={\text{I}}_{4},$$

where I_4_ is the 4 × 4 identity matrix, while to prove (b) one just needs to show that for all *j*, *k* ∈ {1, 2}, we have

$${\text{M}}_{1,j}{\text{M}}_{2,k}={\text{M}}_{2,k}{\text{M}}_{1,j}.$$

Finally, to prove (c), it is enough to show that for all *j*, *k* ∈ {1, 2}, we have

$${\text{I}}_{4}+{\text{M}}_{1,j}{\text{M}}_{2,k}={\text{M}}_{1,j}+{\text{M}}_{2,k}.$$

All this can be shown to be true directly for the matrices (6.6) and (6.7). ▪

Let us now discuss the proven relations. The relation (6.8) can be considered as an alternative check of theorem 5.2. Indeed, since *P*_1_ and *P*_2_ are branch points of order 2 on *S*, the branch 2-lines $L_{\ell }^{\left(j\right)}$ have order 2.

The second relation (6.9) follows from a fundamental property of multi-dimensional complex analysis discussed at the end of §3: the bypasses about $L_{1}^{\left(j\right)}$ and $L_{2}^{\left(k\right)}$ commute.

Finally, the relation (6.10) is discussed in detail in [7]. This relation means that the function *u*_c_ can be represented locally as a sum of two functions having branch 2-lines, separately, at $L_{1}^{\left(j\right)}$ and at $L_{2}^{\left(k\right)}$. As shown in [7,8] the additive crossing property plays a fundamental role in the process of integration of functions of several complex variables.

## 7. Link between finite basis and coordinate equations

Here we continue to study the Dirichlet strip problem from the previous section.

The property (6.5) of the vector W is reminiscent of the behaviour of a Fuchsian ordinary differential equation on a plane of a single complex variable. Namely, the poles of the coefficients of a Fuchsian ODE are branch points of its solution, and the vector composed of linearly independent solutions is multiplied by a constant *monodromy matrix* as the argument bypasses a branch point. Indeed, here the M_ℓ,*j*_ play the role of such monodromy matrices. It is also well known that, conversely, if a vector of linearly independent functions of a single variable has this behaviour, then there exists a Fuchsian equation obeyed by them (this is Hilbert’s 21st problem). This can be shown using the concept of fundamental matrices and their determinants (the Wronskian), see [27].

Here the situation is more complicated. There are two independent complex variables instead of one. Moreover, the behaviour of the components of W at infinity are not explored. However, we can still prove some important statements.

Throughout the paper, it is implicitly assumed that the field *u*, and, thus, its continuation basis W, depend on the angle of incidence φ^in^. Let us now consider four different incidence angles $\varphi_{\text{I}}^{\text{in}}$, $\varphi_{\text{II}}^{\text{in}}$, $\varphi_{\text{III}}^{\text{in}}$ and $\varphi_{\text{IV}}^{\text{in}}$. It hence leads to four different wave fields, and to four basis vectors, all defined by (6.4): W_I_, W_II_, W_III_, W_IV_. Let us construct the 4 × 4 square matrix function V made of these vectors, defined by

7.1 $$\text{V}\equiv ({\text{W}}_{\text{I}},{\text{W}}_{\text{II}},{\text{W}}_{\text{III}},{\text{W}}_{\text{IV}}).$$

We claim here (without proof) that the matrix V is non-singular almost everywhere (in $C^{2}$ minus a set having complex codimension 1), so that we can freely write V^−1^.

Note that the whole matrix is only branching at *T*, and that the equations (6.5) are valid for the matrix V as a whole:

7.2 $$\text{V}\overset{\sigma_{\ell ,j}}{⟶}{\text{M}}_{\ell ,j}\text{V}.$$

This allows us to formulate the following theorem, linking the theory of differential equations to the strip diffraction problem:

**Theorem 7.1.**

*There exist two* 4 × 4 *matrix functions* Z_1_(*x*_1_, *x*_2_) *and* Z_2_(*x*_1_, *x*_2_), *meromorphic in* $C^{2}$, *such that*

(a) *the matrix function* V *satisfies the following differential equations*
  7.3 $$\partial_{x_{1}}\text{V}=\text{V}\;{\text{Z}}_{1}\;\text{ and }\;\partial_{x_{2}}\text{V}=\text{V}\;{\text{Z}}_{2};$$
(b) *these matrix functions obey the consistency relation*:
  7.4 $${\text{Z}}_{1}\;{\text{Z}}_{2}-{\text{Z}}_{2}\;{\text{Z}}_{1}=\partial_{x_{2}}{\text{Z}}_{1}-\partial_{x_{1}}{\text{Z}}_{2},$$

*where* ∂*x*_ℓ_ *for* ℓ ∈ {1, 2} *are the complex derivatives defined in* (3.2).

**Proof.**

For (a) assume that V is known and that V^−1^ exists almost everywhere (in $C^{2}$ minus a set of complex codimension 1). In this case, the coefficients Z_1_ and Z_2_ are simply given by

7.5 $${\text{Z}}_{1}={\text{V}}^{-1}\partial_{x_{1}}\text{V}\;\text{ and }\;{\text{Z}}_{2}={\text{V}}^{-1}\partial_{x_{2}}\text{V}.$$

Let us show that the matrices Z_1_ and Z_2_ are single-valued in $C^{2}$. The only sets at which one can expect branching are the branch 2-lines of V, i.e. $L_{\ell }^{\left(j\right)}$. Make a bypass *σ*_ℓ,*j*_ about a 2-line $L_{\ell }^{\left(j\right)}$ and study the change of Z_1_ and Z_2_ as the result of this bypass:

7.6 $${\text{Z}}_{k}\overset{\sigma_{\ell ,j}}{⟶}{\text{V}}^{-1}{\text{M}}_{\ell ,j}^{-1}\partial_{x_{k}}({\text{M}}_{\ell ,j}\text{V})={\text{Z}}_{k},\;k\in \{1,2\},$$

because M_ℓ,*j*_ are constant matrices. Thus, the coefficients Z_1_ and Z_2_ are not changing at the branch 2-lines of V, and, therefore, they are single-valued in $C^{2}$. A detailed study shows that they have simple polar sets at the lines $L_{\ell }^{\left(j\right)}$, and, possibly, polar sets at the zeros of det(V) though we omit this discussion here for brevity.

To prove (b), differentiate the first equation of (7.3) with respect to *x*_2_, and the second equation with respect to *x*_1_. The expressions on the left are equal, and we get

$$(\partial_{x_{2}}\text{V}){\text{Z}}_{1}+\text{V}\partial_{x_{2}}{\text{Z}}_{1}=(\partial_{x_{1}}\text{V}){\text{Z}}_{2}+\text{V}\partial_{x_{1}}{\text{Z}}_{2}.$$

Applying (7.3) and multiplying by V^−1^, we obtain (7.4).

As it follows from Frobenius theorem, this relation guarantees the solvability of the system (7.3). ▪

A detailed form of the coefficients Z_1_ and Z_2_ can be found in [1–3]. Here our aim was just to demonstrate that the existence of the coordinate equations is connected with the structure of analytical continuation of the solution. It has to be stressed that one can use the coordinate equation to evaluate the physical wave field (see e.g. [1]) and hence, our method, by providing a systematic way of obtaining coordinate equations, can in principle be used to recover the physical field.

Finally, before concluding the paper, it is interesting to note that the idea of considering a set of different incident angles and trying to link the solutions to each other by means of differential equations is somewhat reminiscent of Biggs’ interpretation of embedding formulae [28].

## 8. Conclusion

We have provided an explicit method to analytically continue two-dimensional wave fields emanating from a broad range of diffraction problems and described the singular sets (in $C^{2}$) of their analytical continuation. We have shown that, even though the analytical continuation may have potentially infinitely many branches, each branch can be expressed as a linear combination of finitely many basis functions. Such basis functions are expressed as Green’s integrals over a real double-eight contour. The effectiveness of the general theory was illustrated via the example of diffraction by an ideal strip, for which we proved that only four basis functions were needed. Using these, we were able to completely describe the analytical continuation and study its branching behaviour. Finally, we have shown that this finite basis property was directly related to the existence of the so-called coordinate equation for the strip problem.

## Figures and Tables

**Figure 10.**
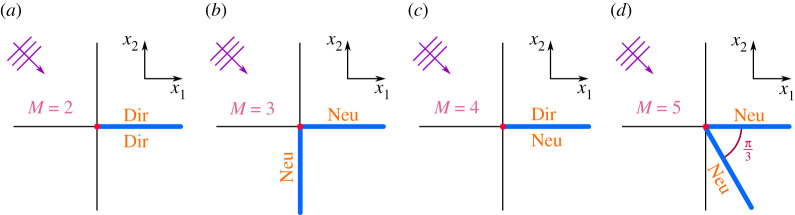
Exampleof scatterers (thick blue lines) with *N* = 1 branch point (thick red dot). Dir-Dir and Dir-Neu half-planes (*a*) and (*c*), and Neu-Neu wedges (*b*) and (*d*). (Online version in colour.)

**Figure 11.**
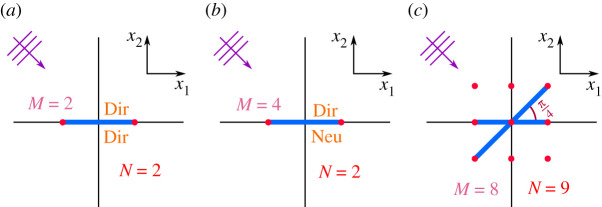
Example of scatterers (thick blue lines) with *N* > 1 branch points(thick red dots). Dir-Dir and Dir-Neu strips (*a*) and (*b*) and cross-like scatterer (*c*). (Online version in colour.)

**Figure 12.**
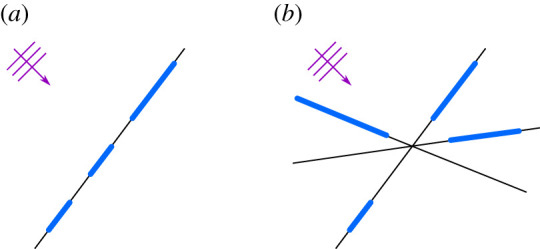
Example of multiple non-intersectingscatterers (thick blue lines), the supporting lines are illustrated in thin black lines. Set of aligned strips (*a*) and a star-shaped arrangement of strips and half-planes (*b*). (Online version in colour.)

## Footnotes

1 To avoid confusion, we remind the reader that (3.1) does imply that *u*_*c*_ is harmonic (i.e. satisfies Laplace’s equation) in the planes (Re[*x*_ℓ_], Im[*x*_ℓ_]) for ℓ = 1, 2, while (3.1) and (3.2) imply that it satisfies the Helmholtz equation in the plane (Re[*x*_1_], Re[*x*_2_]).

## Data accessibility

This article has no additional data.

## Appendix A. Diffraction problems on a plane and on Sommerfeld surfaces

In this appendix, we aim to describe the wide class of two-dimensional diffraction problems for which the theory developed in the paper is valid.

Consider an incident plane wave *u*_in_ impinging on a set of scatterers. The aim is to find the resulting total field *u* satisfying the Helmholtz equation and subject to some specified boundary conditions on the scatterers. For the problem to be well-posed, we also require that *u* have bounded energy at the edges of the scatterers (Meixner condition) and that the scattered field *u* − *u*_in_ be outgoing (radiation condition).

For the theory of the paper to be applicable to such a problem, it should obey the four following simple rules:

R1: all scatterers faces should be straight segments, possibly intersecting, possibly of infinite length;
R2: the boundary conditions should be of Neumann or Dirichlet type, they are allowed to be different on each side of the segment;
R3: the segments should either have the same supporting line or, failing that, their supporting lines should all intersect at one common point;
R4: the angles between the supporting lines of each segment should be rational multiples of *π*.

Provided that R1–R4 are satisfied, the diffraction problem can be reformulated as a propagation problem on a Sommerfeld surface, without scatterers *per se*, but with a finite number *N* of branch points and a finite number *M* of sheets.

One can formalize the procedure of building the Sommerfeld surface *S* and the solution *u* on it provided that the solution *u* in the physical domain is known. Consider the origin of the polar coordinates (*ρ*, φ) to be the intersection point mentioned in the rule R3 (or anywhere on the support line if there is only one). The solution *u* can be continued past the faces of the scatterers by following the reflection equations across each face of the scatterers:

A 1 (Dirichlet): u(ρ,Φ+φ)=−u(ρ,Φ−φ)

and

A 2 (Neumann): u(ρ,Φ+φ)=+u(ρ,Φ−φ),

where (*ρ*, *Φ*) is a point belonging to a scatterer’s face.

In other words, one should take the physical domain with the solution *u* on it, make cuts along each scatterer’s face, make enough copies of the physical plane by reflection across some lines (the supporting lines of the scatterers and of their successive reflections), and attach the obtained reflected planes to each other according to these reflection equations.

*Examples.* As illustrated in figure 10, examples satisfying the conditions R1–R4 with *N* = 1 branch point include: the Dirichlet–Dirichlet (Dir-Dir) or Neumann–Neumann (Neu-Neu) half-plane (*M* = 2), the Dirichlet–Neumann (Dir-Neu) half-plane (*M* = 4) and any Dir-Dir or Neu-Neu wedge with internal angle *pπ*/*q* with $(p,q)\in N_{>0}$ and *q* > *p*, for which *M* is equal to the denominator of the irreducible form of the fraction *q*/(2 *q* − *p*). All these can be solved by the Wiener–Hopf technique, see e.g. [22] for figure 10*a*, [29] for figure 10*d* and [30] for figure 10*b*,*d*.

Examples with higher numbers of branch points, illustrated in figure 11, include the Dir-Dir or Neu-Neu strip (*N* = 2, *M* = 2), the Dir-Neu strip (*N* = 2, *M* = 4), or more complicated scatterers such as two intersecting Dir-Dir strips with respective angles *π*/4 and 3*π*/4, for which branch points not belonging to the physical scatterers start to occur (*N* = 9, *M* = 8, etc).

Finally, configurations with multiple non-intersecting scatterers are also possible, as illustrated in figure 12. The case of multiple aligned strips (figure 12*a*) is a diffraction problem that has previously been investigated in e.g. [1] (via coordinate equations) or [31] (via an iterative Wiener–Hopf method).

## Appendix B. Complex Green’s theorem

In this appendix we aim to formulate a complex version of Green’s theorem. In order to do so we need to introduce the notion of the *complex gradient* of a function of several complex variables. Because of the topic of the paper, it is enough to focus on $C^{2}$, for which it can be defined as follows. For a function *v*(*x*_1_, *x*_2_) of two complex variables *x*_1_ and *x*_2_, the complex gradient of *v*, denoted $\nabla_{C}v$, is defined as the complex 1-form

$$\nabla_{C}v\equiv \partial_{x_{1}}v\;dx_{2}-\partial_{x_{2}}v\;\text{d}x_{1}.$$

The complex Green’s theorem in $C^{2}$ can hence be formulated as follows.

Theorem B.1 (Complex Green’s theorem). *If two functions v* (*x*_1_, *x*_2_) *and w* (*x*_1_, *x*_2_) *both obey the complex Helmholtz equation* (3.2) *in some neighbourhood* (*included in* $C^{2}$) *where they are analytic, i.e. where they satisfy the Cauchy–Riemann conditions* (3.1), *then, in this neighbourhood*,

$$d(v\nabla_{C}w-w\nabla_{C}v)=0,$$

*where d is the usual exterior derivative operator for differential forms.*

Proof. Following [32], it is convenient to decompose the exterior derivative *d* as $d=\partial +\bar{\partial }$, where ∂ and $\bar{\partial }$ are the so-called Dolbeault operators. For any function (0-form) *f* (*x*_1_, *x*_2_), they are defined by

$$\partial f=\partial_{x_{1}}fdx_{1}+\partial_{x_{2}}fdx_{2}\;\text{and}\;\bar{\partial }f=\partial_{{\bar{x}}_{1}}fd{\bar{x}}_{1}+\partial_{{\bar{x}}_{2}}fd{\bar{x}}_{2},$$

while for any complex 1-form $\omega =f_{1}(x_{1},x_{2})dx_{1}+f_{2}(x_{1},x_{2})dx_{2}+g_{1}(x_{1},x_{2})d{\bar{x}}_{1}+g_{2}(x_{1},x_{2})d{\bar{x}}_{2}$, they are given by

$$\begin{array}{rl} \partial \omega & =\partial f_{1}\wedge dx_{1}+\partial f_{2}\wedge dx_{2}+\partial g_{1}\wedge d{\bar{x}}_{1}+\partial g_{2}\wedge d{\bar{x}}_{2},\\ \bar{\partial }\omega & =\bar{\partial }f_{1}\wedge dx_{1}+\bar{\partial }f_{2}\wedge dx_{2}+\bar{\partial }g_{1}\wedge d{\bar{x}}_{1}+\bar{\partial }g_{2}\wedge d{\bar{x}}_{2}. \end{array}$$

Hence, using the fact that *v*, *w* and their derivatives are analytic we can show that $\bar{\partial }(v\nabla_{C}w-w\nabla_{C}v)=0$, leading to

$$\begin{array}{rl} d(v\nabla_{C}w-w\nabla_{C}v) & =\partial (v\nabla_{C}w-w\nabla_{C}v)\\ & =(\partial_{x_{1}}(v\partial_{x_{1}}w)+\partial_{x_{2}}(v\partial_{x_{2}}w)-\partial_{x_{1}}(w\partial_{x_{1}}v)-\partial_{x_{2}}(w\partial_{x_{2}}v))dx_{1}\wedge dx_{2}, \end{array}$$

where we have used that $dx_{\ell }\wedge dx_{\ell }=0$ for ℓ ∈ {1, 2} and that $dx_{1}\wedge dx_{2}=-dx_{2}\wedge dx_{1}$. Upon expanding out the derivatives, we obtain

$$d(v\nabla_{C}w-w\nabla_{C}v)=(v(\partial_{x_{1}}^{2}w+\partial_{x_{2}}^{2}w)-w(\partial_{x_{1}}^{2}v+\partial_{x_{2}}^{2}v))dx_{1}\wedge dx_{2}=0,$$

as required, since both *v* and *w* satisfy the complex Helmholtz equation. ▪

Note that the same result can be proven in $C^{3}$. In order to do so, one has to define the complex gradient of a function *v*(*x*_1_, *x*_2_, *x*_3_) as

$$\nabla_{C}v=\partial_{x_{1}}v\;dx_{2}\wedge dx_{3}+\partial_{x_{2}}v\;dx_{3}\wedge dx_{1}+\partial_{x_{3}}v\;dx_{1}\wedge dx_{2}.$$

These complex gradients definitions are closely related to and can be expressed in terms of the Hodge star operator.

The complex Green’s theorem, combined with Stokes’ theorem for complex differential forms, makes it possible to show that for any two functions *v* and *w* satisfying the hypotheses of theorem B.1, and two contours *γ*_1_ and *γ*_2_ that can be deformed homotopically to each other within the region of analyticity of *v* and *w*, we have

B 1 $$\int_{\gamma_{1}}(v\nabla_{C}w-w\nabla_{C}v)=\int_{\gamma_{2}}(v\nabla_{C}w-w\nabla_{C}v).$$

Hence, the value of the integral is not changed when the contour is deformed, even within $C^{2}$. Moreover, note that when restricted to a contour *γ* in $R^{2}$, we have

$$v\nabla_{C}w-w\nabla_{C}v=\left[v\frac{\partial w}{\partial n}-w\frac{\partial v}{\partial n}\right]\text{d}l.$$

The link with what we have done in the paper becomes clear by choosing *v* ≡ *u*_*c*_ and *w* ≡ *G*.

To prove that *u*_*c*_(*A*) is indeed defined uniquely by (4.1) in a small complex neighbourhood of *A*_0_, proceed as follows.^2^ Let $A\equiv (x_{1}^{A},x_{2}^{A})\in C^{2}[x_{1},x_{2}]\setminus T$ in a small neighbourhood of *A*_0_ ∈ *S*, and let *u*_*c*_(*A*) be the value obtained by letting **r** become a complex vector, and choosing *A* close enough to *A*_0_ such that *G*(**r**, **r**′) remains regular for **r**′ ∈ *γ*.

Consider now the change of variable $\left(\xi_{1},\xi_{2})=(x_{1}-x_{1}^{A},x_{2}-x_{2}^{A}\right)$ so that in $C^{2}[\xi_{1},\xi_{2}]$, we have *A* ≡ (0, 0), so that $A\in C^{2}[\xi_{1},\xi_{2}]\cap R^{2}[\xi_{1},\xi_{2}]$, and *u*_*c*_ can be studied as a function obeying the Helmholtz equation on the real plane $R^{2}[\xi_{1},\xi_{2}]$. This can be done by considering the function ${\tilde{u}}_{c}(\xi_{1},\xi_{2})=u_{c}(\xi_{1}+x_{1}^{A},\xi_{2}+x_{2}^{A})$, and, as such, it is given by Green’s formula

$$u_{c}(A)={\tilde{u}}_{c}(0,0)=\int_{\tilde{\gamma }}\left[{\tilde{u}}_{c}({\tilde{\text{r}}}^{\prime })\frac{\partial G}{\partial n^{\prime }}(\text{0},{\tilde{\text{r}}}^{\prime })-G(\text{0},{\tilde{\text{r}}}^{\prime })\frac{\partial {\tilde{u}}_{c}}{\partial n^{\prime }}({\tilde{\text{r}}}^{\prime })\right]\text{d}{\tilde{l}}^{\prime }=\int_{\tilde{\gamma }}(u_{c}\nabla_{C}G-G\nabla_{C}u_{c}),$$

where $\tilde{\gamma }$ is a real contour encircling the origin of $R^{2}[\xi_{1},\xi_{2}]$, ${\tilde{\text{r}}}^{\prime }$ is a real vector pointing to a point in $\tilde{\gamma }$ and **0** is the zero vector. The last equality comes from the fact that a form is independent of the coordinate system in which it is expressed, and from the translational invariance of *G* that satisfies $G(x_{1}-x_{1}^{A},x_{2}-x_{2}^{A};x_{1}^{\prime }-x_{1}^{A},x_{2}^{\prime }-x_{2}^{A})=G(x_{1},x_{2};x_{1}^{\prime },x_{2}^{\prime })$.

Now when viewed in $C^{2}[x_{1},x_{2}]$, $\tilde{\gamma }$ is not a contour in $R^{2}[x_{1},x_{2}]$, but by (B 1) we can deform this contour to the contour $\gamma \subset R^{2}[x_{1},x_{2}]$ used in (4.1) without changing the value of the integral to get

$$u_{c}(A)=\int_{\gamma }(u_{c}\nabla_{C}G-G\nabla_{C}u_{c})=\int_{\gamma }\left[u_{c}({\text{r}}^{\prime })\frac{\partial G}{\partial n^{\prime }}(\text{r},{\text{r}}^{\prime })-G(\text{r},{\text{r}}^{\prime })\frac{\partial u_{c}}{\partial n^{\prime }}({\text{r}}^{\prime })\right]\text{d}l^{\prime },$$

as expected, where **r′** is a real vector pointing to *γ* and **r** is a complex vector pointing to *A*, which is exactly what we would have obtained by letting **r** become complex in (4.1). The contours used and the subsequent deformation are illustrated in figure 4.

## Appendix C. Bypasses and argument of the Hankel function

Let $A=(x_{1},x_{2})\in C^{2}\setminus (T\cup R^{2})$. We are interested in the behaviour of the function

$$G(x_{1}^{\prime },x_{2}^{\prime })=H_{0}^{\left(1\right)}\left(k\sqrt{{\left(x_{1}-x_{1}^{\prime }\right)}^{2}+{\left(x_{2}-x_{2}^{\prime }\right)}^{2}}\right),$$

in the real plane $(x_{1}^{\prime },x_{2}^{\prime })\in R^{2}$. In particular we want to know how this function changes along a given contour *Γ* within this real plane. Upon introducing the two fixed complex quantities *z*_1_ = *x*_1_ + *i x*_2_ and *z*_2_ = *x*_1_ − *i x*_2_ we can rewrite G as

$$G(x_{1}^{\prime },x_{2}^{\prime })=H_{0}^{\left(1\right)}\left(k\sqrt{\left[z_{1}-(x_{1}^{\prime }+ix_{2}^{\prime })][z_{2}-(x_{1}^{\prime }-ix_{2}^{\prime })\right]}\right).$$

For this purpose, it is also convenient to see the real (*x*_1_′, *x*_2_′) real plane as a complex plane of the one complex variable *z*′ = *x*_1_′ + *ix*_2_′, so that G can be considered as a function of the complex variable *z*′ and we may write

$$G(z^{\prime })=H_{0}^{\left(1\right)}\left(k\sqrt{[z^{\prime }-z_{1}]\bar{\left[z^{\prime }-\bar{z_{2}}\right]}}\right).$$

In the *z*′ complex plane, the points *A*_1_ and *A*_2_ are defined as the points *z*′ = *z*_1_ and $z^{\prime }=\bar{z_{2}}$, respectively.

Consider now a contour fragment *Γ* that encircles the point *A*_1_, i.e. the point *z*′ = *z*_1_, once in the anticlockwise direction. It does not encircle the point *A*_2_. Upon translating the origin of the *z*′ plane by considering the new variable *ξ*′_1_ = *z*′ − *z*_1_, we can rewrite *G* in the *ξ*′_1_ plane as

$$G(\xi_{1}^{\prime })=H_{0}^{\left(1\right)}\left(k\sqrt{\xi_{1}^{\prime }\bar{\left[\xi_{1}^{\prime }+z_{1}-\bar{z_{2}}\right]}}\right).$$

In the *ξ*′_1_ plane, *Γ* encircles 0 once anticlockwise, but does not encircle $-z_{1}+\bar{z_{2}}$, so that if we parametrize *Γ* by *τ* ∈ [0, 1], we have

$$\begin{array}{rl} \xi_{1}^{\prime }(0) & \overset{\Gamma }{\to }\xi_{1}^{\prime }(1)=e^{2i\pi }\xi_{1}^{\prime }(0),\\ \bar{\left[\xi_{1}^{\prime }(0)+z_{1}-\bar{z_{2}}\right]} & \overset{\Gamma }{\to }\bar{\left[\xi_{1}^{\prime }(1)+z_{1}-\bar{z_{2}}\right]}=\bar{\left[\xi_{1}^{\prime }(0)+z_{1}-\bar{z_{2}}\right]}. \end{array}$$

Hence, if one introduces the argument of the Hankel function to be

$$Z=k\sqrt{\xi_{1}^{\prime }\bar{\left[\xi_{1}^{\prime }+z_{1}-\bar{z_{2}}\right]}},$$

we have

$$Z(0)=k\sqrt{\xi_{1}^{\prime }(0)\bar{\left[\xi_{1}^{\prime }(0)+z_{1}-\bar{z_{2}}\right]}}\overset{\Gamma }{\to }k\sqrt{e^{2i\pi }\xi_{1}^{\prime }(0)\bar{\left[\xi_{1}^{\prime }(0)+z_{1}-\bar{z_{2}}\right]}}=e^{i\pi }Z(0).$$

Hence, when a fragment of a contour encircles *A*_1_ once in the anticlockwise direction, the argument of the Hankel function is indeed changed from $Z\to e^{i\pi }Z$. Naturally if the bypass was clockwise, the argument would pass from $Z\to e^{-i\pi }Z$.

A similar argument, introducing the new variable $\xi_{2}^{\prime }=z^{\prime }-\bar{z_{2}}$, leads to the fact that when a fragment of a contour encircles *A*_2_ once in the anticlockwise direction, the argument of the Hankel function is indeed changed from $Z\to e^{-i\pi }Z$. Again if the bypass was clockwise, the argument would pass from $Z\to e^{i\pi }Z$.
